# Metal-Free Air Oxidation in a Convenient Cascade Approach for the Access to Isoquinoline-1,3,4(*2H*)-triones

**DOI:** 10.3390/molecules24112177

**Published:** 2019-06-10

**Authors:** Antonia Di Mola, Consiglia Tedesco, Antonio Massa

**Affiliations:** Dipartimento di Chimica e Biologia “A. Zambelli”, Università degli studi di Salerno, Via Giovanni Paolo II, 84084-Fisciano, Salerno, Italy; ctedesco@unisa.it

**Keywords:** heterocycles, metal-free oxidation, one-pot reactions

## Abstract

Herein we describe a very useful application of the readily available trifunctional aromatic ketone methyl-2-(2-bromoacetyl)benzoate in reactions with primary amines. An unexpected in situ air oxidation that follows a cascade process allowed the access to a series of isoquinoline-1,3,4(*2H*)-triones, a class of heterocyclic compounds of great interest containing an oxygen-rich heterocyclic scaffold. A modification of the original protocol, utilizing a Staudinger reaction in the presence of trimethylphosphine, was necessary for the synthesis of Caspase inhibitor trione with free NH group.

## 1. Introduction

**Scheme 1:**
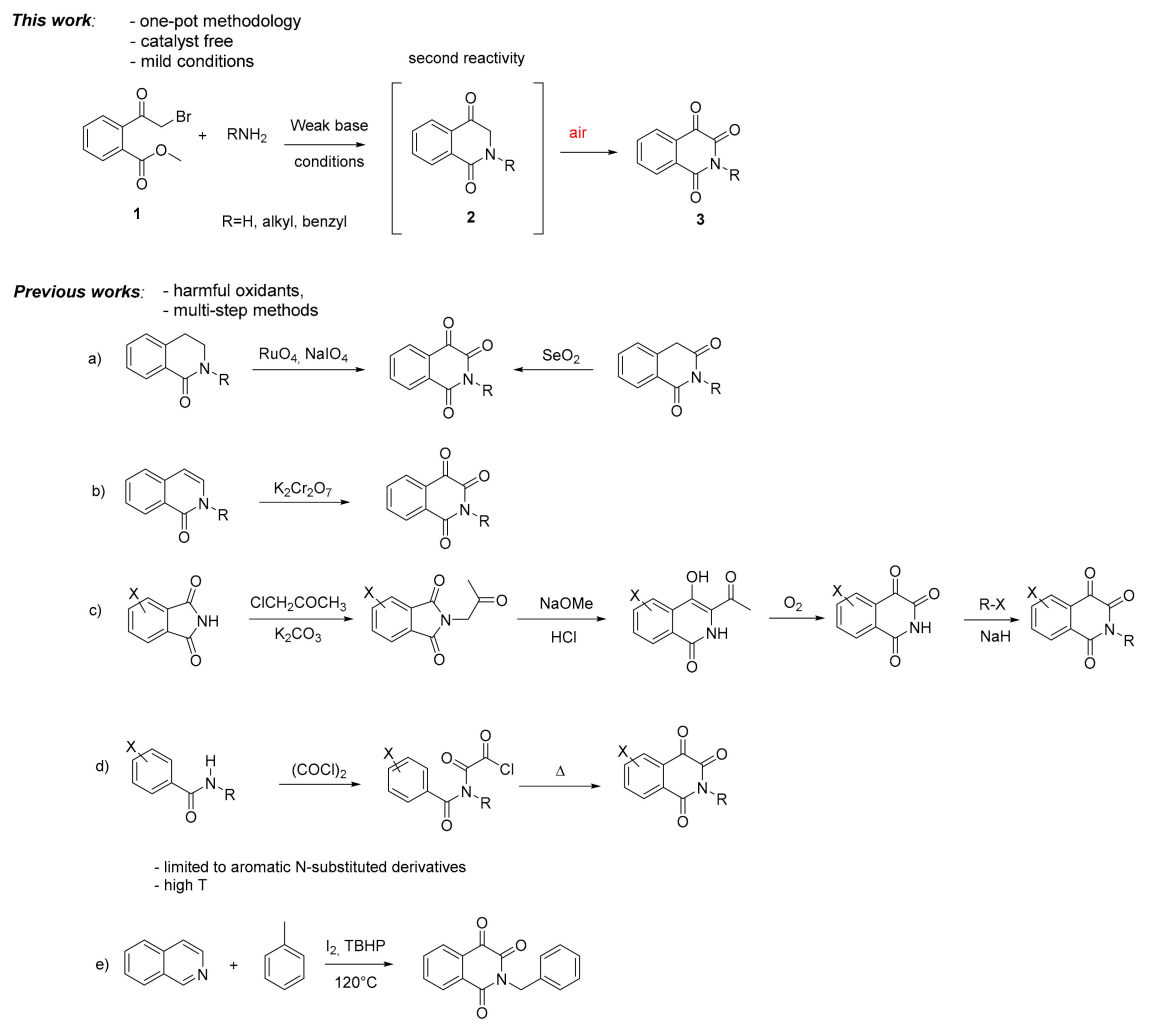
This work and literature methodologies for the synthesis of 2,3-dihydroisoquinoline-1,3,4-trione.

The interest of academia and industry toward one-pot reactions has notably increased during the last two decades because of the environmentally benign conditions deriving from the high level of atom and step economy [1]. These transformations, which also include cascade, tandem, and domino reactions, generally refer to a process involving two or more consecutive reactions, without the isolation of the intermediates. The development of effective one-pot reactions is related to the rational design of multifunctional starting materials [2]. Within this context, we have recently investigated the reactivity as electrophiles in cascade reactions of easily accessible bifunctional aromatic ketones like 2-acylbenzonitriles [3,4,5], developing a particularly convenient process for the synthesis of highly functionalized isoindolinones [3,4,5], a valuable class of heterocyclic compounds [6]. Continuing the efforts to explore the chemistry of multifunctional ketones, we were interested to investigate the reactivity of readily available methyl 2-(2-bromoacetyl)benzoate **1** with primary amines (Scheme 1). There are relatively few reports on the applications of methyl 2-(2-bromoacetyl)benzoate, which consist of displacement reactions with thiols for the synthesis of anticancer agents 1,4-disubstituted phthalazines [7,8] or the preparation of Gd(III)-complexes involving reactions with secondary amines [9]. In this project, we have focused on the possibility to obtain in a single pot procedure 2,3-dihydroisoquinoline-1,4-diones **2** and to study the second reactivity for instance in oxidation reactions, which can allow the access to 2,3-dihydroisoquinoline-1,3,4-triones **3** (Scheme 1).

**Figure 1:**
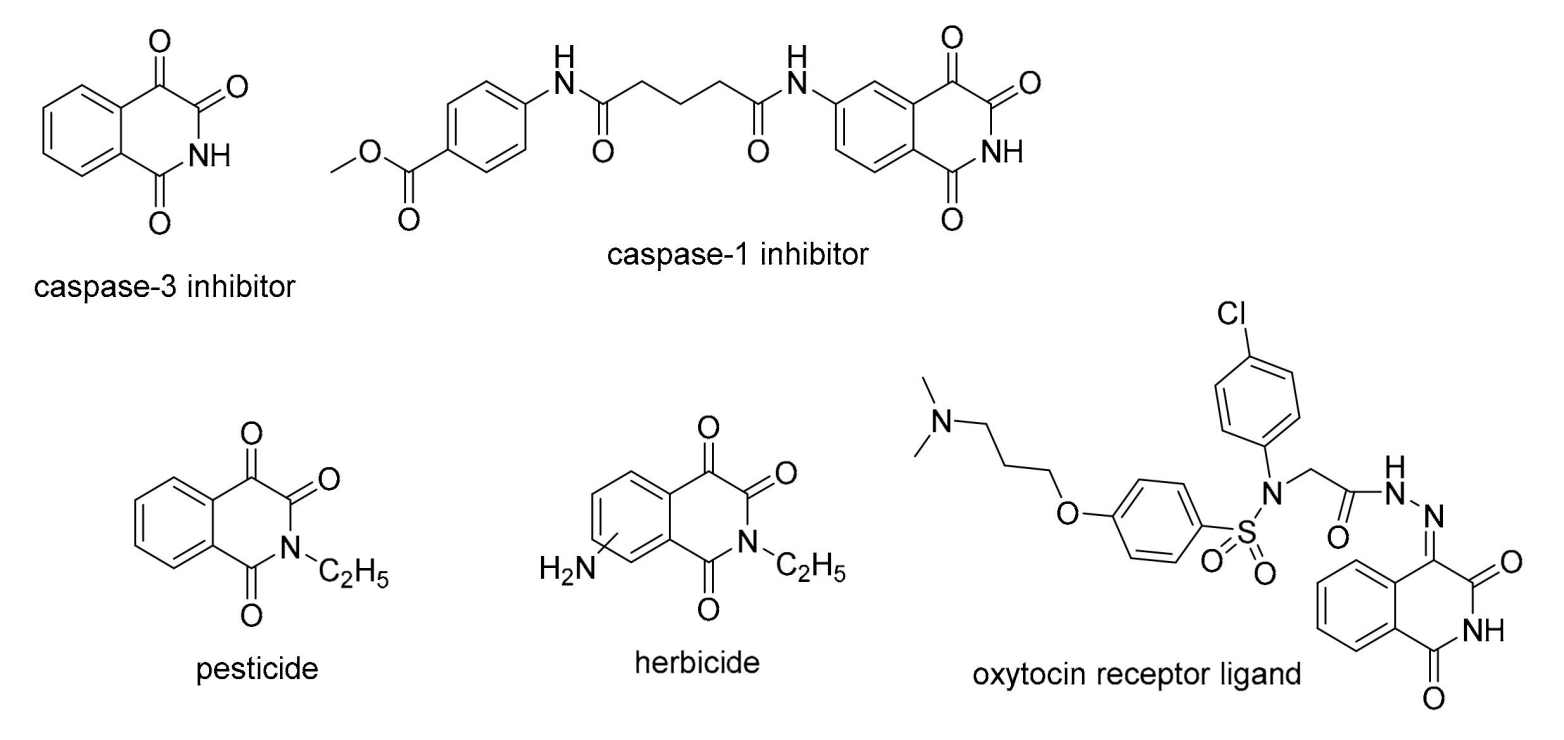
1,3,4(*2H*)-Isoquinolinetrione derivatives of biological interest.

2,3-Dihydroisoquinoline-1,3,4-triones **3** are highly appealing for the number of biological activities as caspase inhibitors [10,11,12], nerve protectors to treat various neurodegenerative diseases, especially Alzheimer’s disease apoplexy and ischemic brain injuries [12,13]. Amino-substituted derivatives have been found to be active as herbicides due to their efficient redox mediation of photosystem I and their activity was found to be greater than that of the parent trione (Figure 1) [14,15,16,17]. The semicarbazide derivative prepared from phthalonimide shows good binding affinity toward oxytocin receptors (*Ki* = 1.6 nM) [18]. In addition, these compounds are particularly useful as intermediates in the synthesis of biologically active alkaloids such as benzo[c]phenanthridine alkaloids or nuevamine [19,20]. In contrast to their fascinating biological profiles, the methods for synthesis of these compounds are relatively limited, usually requiring multi-step sequences employing harsh conditions or harmful reagents with tedious purifications of the intermediates or show a limited substrate scope (Scheme 1).

One of the first synthesis approach was carried out by the oxidation of 3,4-dihydroisoquinolin-1,3-dione with RuO_4_ as a catalyst and NaIO_4_ as the stoichiometric oxidant (Scheme 1a) [21]. Later modifications included the oxidation by SeO_2_ (Scheme 1a) [10], tetraphenylporphyrin sensitized photooxygenation (Scheme 1a) [22,23] or the oxidation of C5-substituted isoquinolin-1-one by K_2_Cr_2_O_7_ (Scheme 1b) [10]. Phthalimides can be transformed into isoquinolinetriones after alkylation with chloroacetone and ring expansion in the presence of excess of sodium methoxide to give 4-hydroxyisocarbostyrils. These intermediates in a suitable solvent in the presence of oxygen undergo to an oxidative deacylation (Scheme 1c) [12]. Some isoquinolinetriones can be prepared from the corresponding secondary benzamides, as shown in Scheme 1d: The reactions of appropriate benzamides and oxalyl chloride lead to *N*-aroyloxamoyl chlorides. These isolable intermediates undergo cyclization upon heating when the group X is an activating group such as an alkoxy substituent [24]. Besides the above reactions involving the oxidation of de-aromatized isoquinolines, isoquinoline-1,3,4-trione can also be prepared by the Beckmann rearrangement or the azido-Schmidt reaction of ninhydrin, using hydroxylamine hydrochloride [25] or trimethylsilyl azide [26], respectively. Recently, a more direct access to dihydroisoquinoline-1,3,4(2H)-triones has been reported using I_2_/TBHP as oxidant, but it suffers from a limited substrate scope and harsh reaction conditions (Scheme 1e) [27]. Even though isoquinoline-1,3-diones are known compounds both as intermediates in the synthesis of 2,3-dihydroisoquinoline-1,3,4-triones and bioactive molecules [28,29,30,31,32,33], to the best of our knowlwdge, the analogues 2,3-dihydroisoquinoline-1,4-diones **2** have never been reported in literature. Therefore, the development of an efficient method to prepare 2,3-dihydroisoquinolines-1,4-diones from readily available sources and investigation of their reactivity would be highly desirable.

## 2. Results

**Figure 2:**
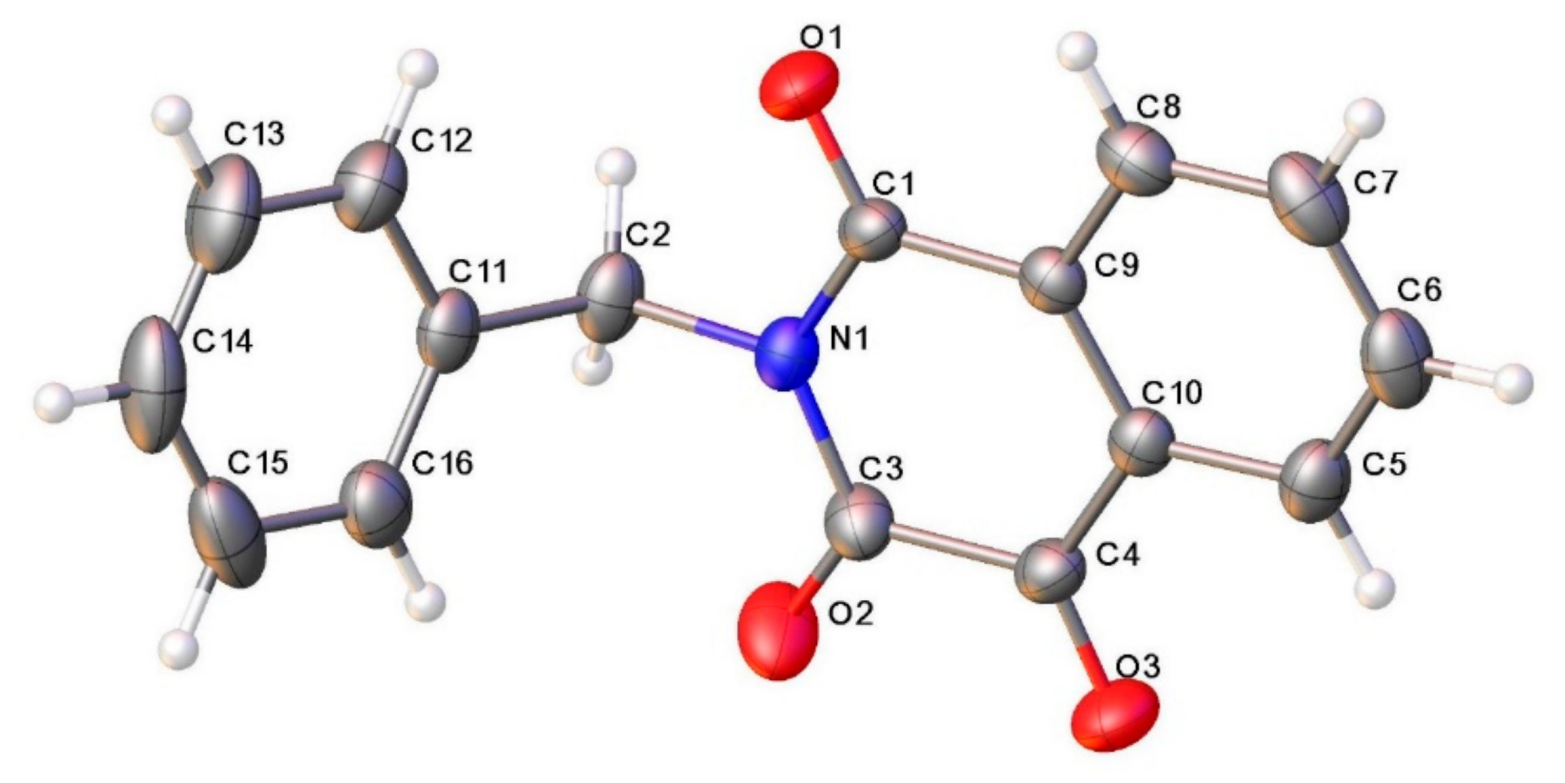
ORTEP drawing and atom numbering scheme of compound **3a**. Hydrogen atoms have been omitted for clarity. Ellipsoids are drawn at 30% probability level. CCDC 1915668 contains the supplementary crystallographic data for this paper. These data can be obtained free of charge via http://www.ccdc.cam.ac.uk/conts/retrieving.html (or from the CCDC, Cambridge, UK).

In order to develop a direct access to 2,3-dihydroisoquinolines-1,4-diones **2**, we treated 2-(2-bromoacetyl)benzoate **1** with one equivalent of benzyl amine, used as model nucleophile, at room temperature in dry CH_3_CN (Table 1). However, under the conditions reported in Entries 1 and 2 of Table 1, the unreacted starting materials were recovered. The conversion was significantly improved in the presence of one equivalent of diisopropylethylamine (DIPEA) at 50 °C (Entry 3–5). In this case, instead of the expected 2,3-dihydroisoquinoline-1,4-dione **2**, we isolated the respective 2,3-dihydroisoquinoline-1,3,4-trione **3a** in a very good 71% yield (Entry 5). The structure of the obtained product was clearly attributed by single-crystal X-ray diffraction, MS spectrum, and comparison of ^1^H-NMR spectrum with literature [27]. The X-ray molecular structure is reported in Figure 2 (for details check Supplementary Materials).

In a control experiment performed under nitrogen atmosphere at 50 °C (Entry 6), we observed the formation of a mixture of products, and only a tiny amount of **3a** was isolated after chromatography. Since ^1^H-NMR performed on the crude did not highlight the presence of **3a,** its formation was probably due to slow air oxidation during purification and isolation procedures (Entry 6). We also tested other bases. However, either Et_3_N or inorganic bases like NaHCO_3_ and K_2_CO_3_ were less effective, leading to decomposition products in a larger extent (Entries 7–11).

Then, under the optimized reaction conditions, we briefly analyzed the scope of the method using a series of aliphatic and benzyl primary amines (Table 2). In all the cases, also with the highly volatile methylamine or ethylamine, added as THF solutions, as well as in the presence of benzyl amines substituted with both electron-withdrawing or -donating groups, we obtained the 2,3-dihydroisoquinoline-1,3,4-triones in good yields. Only in the presence of the less nucleophilic aniline, even though after 18 h at 50 °C the starting materials disappeared, we recovered a complex mixture of products (Entry 10).

**Scheme 2:**
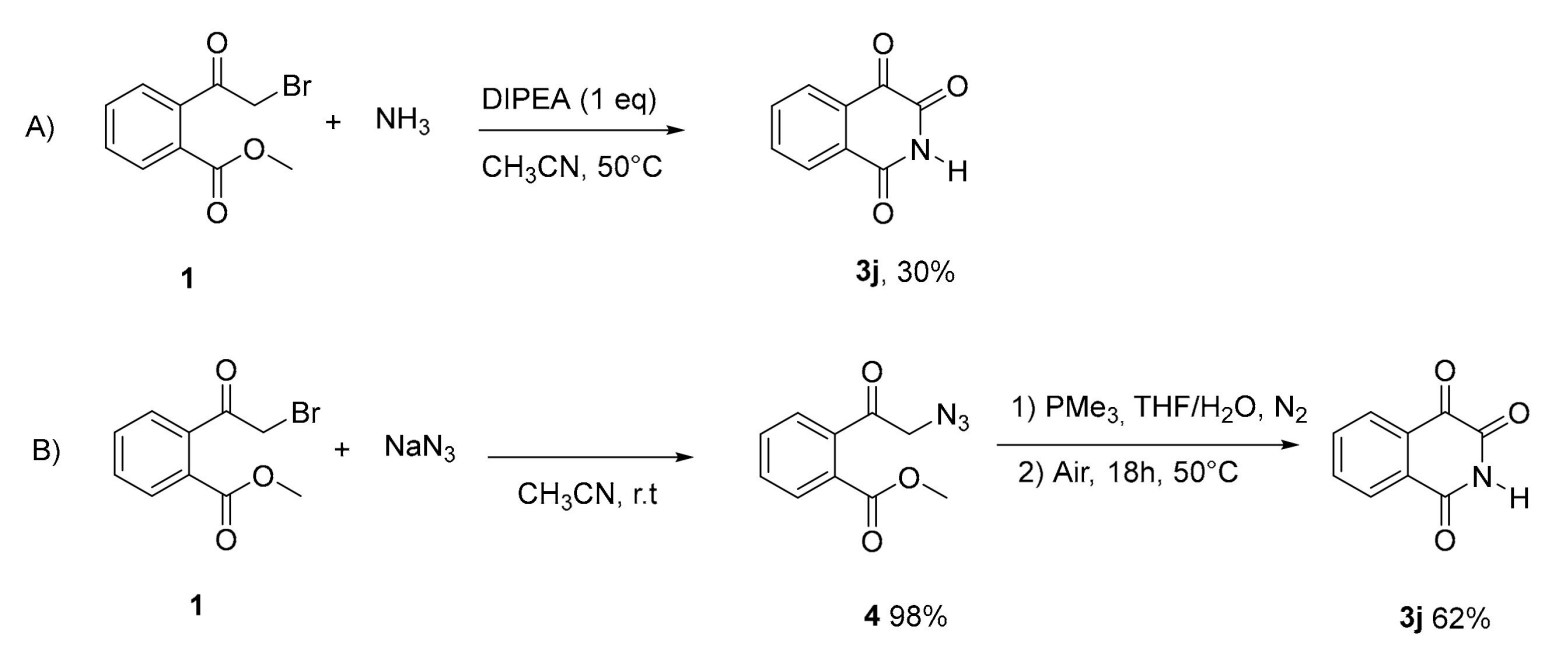
Synthesis of NH-unsubstitued 2,3-dihydroisoquinoline-1,3,4-trione.

We also investigated the reaction of **1** with ammonia, used as 0.5 M solution in dioxane in order to developed one-pot protocol in the synthesis of the 3-caspase inhibitor [12]. The reaction, as summarized in Scheme 2A, was sluggish. The expected product was obtained only in 30% yield together with a series of not well identified intermediates and decomposition products. To this purpose, we modified the original protocol, reacting **1a** with NaN_3_ and the crude derivative was subjected to Staudinger reaction [34] in the presence of PMe_3_ [35]. Even though the isolation and purification of the azide intermediate was necessary in this case, the reduction, cyclization, and air oxidation were carried out in a single pot reaction, simply changing N_2_ with air at the end of the cascade reaction and reacting for further 18 h at 50 °C. This furnished the expected **3j** in a satisfying 62% yield.

**Scheme 3:**
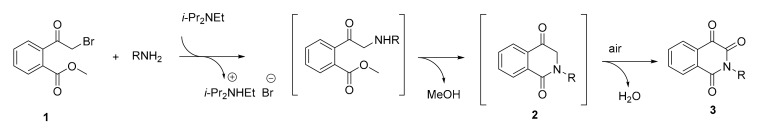
Proposed one-pot mechanism for the developed process.

**Scheme 4:**
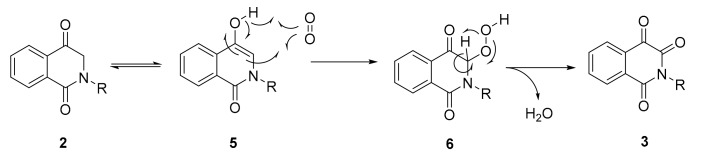
Proposed mechanism for air oxidation to afford 2,3-dihydroisoquinoline-1,3,4-triones.

On the basis of the obtained results and the control experiments, we can confidently propose the mechanism as described in Scheme 3. In particular, we believe that the first steps of the process involve a nucleophilic displacement of bromine and then lactamization. In this sequence of reactions, one equivalent of hindered DIPEA is necessary to neutralize the formed HBr, while the less-hindered Et_3_N could give competing nucleophilic displacement. Once formed the supposed 2,3-dihydroisoquinoline-1,4-diones intermediate **2**, this is subject to in situ air auto-oxidation probably via enol formation **5** (Scheme 4).

Aerobic oxidations without the use of any reagent or metal catalyst are quite rare, but two notable examples have been recently reported for the aerobic α-oxidation of nitrogen containing heterocycles [36,37]. In particular, Foss et al. described an isoindolinone synthesis by selective metal-free aerobic oxidation of isoindolines [36]. In addition, Tang et al. reported an aerobic hydroxylation of isoquinoline-1,3-diones to 4-hydroxy-isoquinoline-1,3-diones by air oxidation via enol formation [37]. Supported by these studies, the enol **5** may react with oxygen in the air, with a following protonation to give hydroperoxide **6**. Then, water elimination furnishes the 2,3-dihydroisoquinoline-1,3,4-triones **3**.

## 3. Materials and Methods

### 3.1. General Information

Reactions were performed using commercially available compounds without further purification and analytical grade solvents. All the reactions were monitored by thin layer chromatography (TLC) on precoated silica gel plates (0.25 mm) and visualized by fluorescence quenching at 254 nm. Flash chromatography was carried out using silica gel 60 (70–230 mesh, Merck, Darmastdt, Germany). The NMR spectra were recorded on Bruker (Rheinstetten, Germany) 400 and 300 spectrometers (400 MHz, ^1^H, 100 MHz, ^13^C, 300 MHz, ^1^H, 75 MHz, ^13^C). Spectra were referenced to residual CHCl_3_ (7.26 ppm, ^1^H, 77.00 ppm, ^13^C). The following abbreviations are used to indicate the multiplicity in NMR spectra: s—singlet, d—doublet, t—triplet, q—quartet, dd—double doublet, ddd—doublet of doublet of doublet, m—multiplet, bs—broad signal. Coupling constants (*J*) are quoted in hertz. Yields are given for isolated products showing one spot on a TLC plate and no impurities detectable in the NMR spectrum. FTIR spectra were recorded as thin films on KBr plates using Bruker (Rheinstetten, Germany) VERTEX 70 spectrometer and absorption maxima are reported in wavenumber (cm^−1^). High-resolution mass spectra (HRMS) were acquired using a Bruker solariX XR Fourier transform ion cyclotron resonance mass spectrometer (Bruker Daltonik GmbH, Bremen, Germany) equipped with a 7T refrigerated actively shielded superconducting magnet. The samples were ionized in a positive ion mode using an electrospray (ESI) ionization source. Methyl 2-(bromoacetyl)benzoate was prepared according to the literature procedure [7].

### 3.2. General Procedure for Isoquinoline-1,3,4(2H)-triones Synthesis

Methyl 2-(bromoacetyl)benzoate (0.24 mmol, 1 equiv.) was dissolved in dry CH_3_CN (2 mL) and DIPEA (1 equiv.) was added followed by the addition of amine (1 equiv.). The mixture was vigorously stirred under air at 50 °C overnight in a round bottomed flask with a rubber stopper and a needle. The solvent was removed under vacuum and the crude was purified by flash column chromatography (Hexane 90/Ethyl acetate 10) using silica gel deactivated with 1% Et_3_N.

### 3.3. Product Characterization

*2-benzylisoquinoline-1,3,4(2H)-trione* (**3a**) [10,21,27,38]: Light yellow solid, 71% yield (45 mg). Mp. 190– 191 °C. Data in accordance with literature. ^1^H-NMR (400 MHz, CDCl_3_) δ 8.36 (dd, *J* = 1.0, 7.8 Hz, 1H), 8.20 (dd, *J* = 7.6, 1.0 Hz, 1H), 7.88 (td, *J* = 1.4, 7.6 Hz, 1H), 7.81 (dt, *J* = 1.3, 7.6 Hz, 1H), 7.51–7.49 (m, 2H), 7.35–7.27 (m, 3H), 5.24 (s, 2H). ^13^C-NMR (100 MHz, CDCl_3_) δ 174.6, 162.0, 156.8, 136.1, 135.9, 134.4, 130.8, 129.8, 129.7, 129.3, 128.6, 127.7, 127.3, 44.3. HRMS (ESI): calcd. for [M + H]^+^ C_16_H_12_NO_3_: 266.0812, found: 266.0814.

*2-(4-chlorobenzyl)isoquinoline-1,3,4(2H)-trione* (**3b**) [27]: Light yellow solid, 73% yield (52 mg). Mp. 187–188 °C. *Data in accordance with literature.* ^1^H-NMR (300 MHz, CDCl_3_) δ 8.35 (d, *J* = 7.7 Hz, 1H), 8.22 (d, *J* = 7.6 Hz, 1H), 7.91 (td, *J* = 1.2, 7.6 Hz, 1H), 7.84 (td, *J* = 1.2, 7.6 Hz, 1H), 7.46 (d, *J* = 8.4 Hz, 2H), 7.28 (d, *J* = 8.4 Hz, 2H), 5.20 (s, 2H). ^13^C-NMR (75 MHz, CDCl_3_) δ 174.7, 162.4, 157.3, 136.7, 134.5, 134.0, 134.3, 131.2, 130.9, 130.8, 129.0, 128.8, 128.1, 43.6. HR-MS (ESI): calcd. for [M + H]^+^ C1_6_H_11_ ClNO_3_: 300.0422, found: 300.0427.

*2-(4-methoxybenzyl)isoquinoline-1,3,4(2H)-trione* (**3c**) [27]: Light yellow solid, 75% yield (57 mg). Mp. 163–164 °C. Data in accordance with literature. ^1^H-NMR (CDCl_3_, 300 MHz) *δ* 8.33 (dd, *J=* 7.4 Hz, 1H), 8.17 (dd, *J =* 7.5 Hz, 1H), 7.91–7.82 (m, 2H), 7.47 (d, *J =* 8.5 Hz, 2H), 6.82 (d, *J =* 8.3 Hz, 2H), 5.15 (s, 2H), 3.76 (s, 3H). ^13^C-NMR (CDCl_3_, 100 MHz) *δ* 174.8, 162.3, 159.5, 156.8, 135.9, 134.3, 131.3, 130.6, 130.1, 129.7, 128.1, 127.6, 113.8, 55.1, 43.6. HR-MS (ESI): calcd. for [M + Na]^+^ C_17_H_13_NO_4_Na: 318.0736, found: 318.0733.

*2-(2-fluorobenzyl)isoquinoline-1,3,4(2H)-trione* (**3d**) [12]: Light yellow solid, 69% yield (47 mg). Mp. 186–187 °C. Data in accordance with literature. ^1^H-NMR (CDCl_3_, 400 MHz) δ 8.36 (dd, *J* = 0.8, 7.8 Hz, 1H), 8.25 (dd, *J* = 0.8, 7.8 Hz, 1H), 7.94–7.82 (m, 2H), 7.34–7.31 (m, 1H), 7.29–7.25 (m, 1H), 7.09–7.04 (m, 2H), 5.36 (s, 2H). ^13^C-NMR (CDCl_3_, 75 MHz) δ 174.7, 162.0 (*J =* 240 Hz), 161.9, 156.9, 136.3, 134.5, 130.8, 130.1 (*J =* 3.0 Hz), 130.0, 129.7, 129.6 (*J =* 9.0 Hz), 127.9, 124.1 (*J =* 3.0 Hz), 122.6 (*J =* 9.0 Hz), 115.6 (*J =* 9.0 Hz), 38.3 (*J =* 3.0 Hz). HR-MS (ESI): calcd. for [M + H]^+^ C_16_H_11_FNO_3_: 284.1707, found: 284.1710.

*2-(3-nitrobenzyl)isoquinoline-1,3,4(2H)-trione* (**3e**) [27]: Light yellow solid, 69% yield (51 mg). Mp. 186–187 °C. Data in accordance with literature. ^1^H-NMR (CDCl_3_, 400 MHz) δ 8.38 (t, *J* = 7.7 Hz, 2H), 8.24 (d, *J* = 7.6 Hz, 1H), 8.16 (*J* = 8.0 Hz, 1H), 7.97–7.94 (m, 1H), 7.89–7.82 (m, 2H), 7.52 (t, *J* = 8.0 Hz, 1H), 5.34 (s, 2H). ^13^C-NMR (100 MHz, CDCl_3_) δ 174.7, 162.3, 157.1, 148.8, 137.5, 136.4, 135.2, 135.9, 131.3, 130.3, 129.9, 129.8, 128.2, 124.5, 123.4, 43.8. HR-MS (ESI): calcd. for [M + H]^+^ C_16_H_11_N_2_O_5_: 311.0662, found: 311.0659.

*2-Methylisoquinoline-1,3,4(2H)-trione* (**3f**) [10,12,21,38]: Light yellow solid, 72% yield (32 mg). Mp. 186–187 °C. Data in accordance with literature**.** ^1^H-NMR (CDCl_3_, 300 MHz) δ 8.36 (dd, *J* = 1.3, 7.6 Hz, 1H), 8.23 (dd, *J* = 1.3, 7.6 Hz, 1H), 7.94–7.81 (m, 2H), 3.50 (s, 3H). ^13^C-NMR (CDCl_3_, 100 MHz) δ 174.5, 162.4, 157.3, 136.20, 134.4, 130.6, 129.8, 129.7, 127.8, 27.5. HR-MS (ESI): calcd. for [M + H]^+^ C_10_H_8_NO_3_: 190.0499, found: 190.0492.

*2-Ethylisoquinoline-1,3,4(2H)-trione* (**3g**) [14,21,38,39]: Light yellow solid, 70% yield (34 mg). Mp. 101–102 °C. Data in accordance with literature. ^1^H-NMR (CDCl_3_, 300 MHz) δ 8.38 (dd, *J* = 0.6, 5.8 Hz, 1H), 8.24 (dd, *J* = 0.8, 5.8 Hz, 1H), 7.94–7.83 (m, 2H), 4.15 (q, *J* = 7.1 Hz, 2H), 1.28 (t, *J* = 7.1 Hz, 3H). ^13^C-NMR (CDCl_3_, 100 MHz) δ 174.6, 161.8, 156.7, 135.9, 134.3, 130.7, 129.9, 129.7, 127.7, 37.3, 13.1. HR-MS (ESI): calcd. for [M + H]^+^ C_11_H_10_NO_3_: 204.0655, found: 204.0658.

*2-Butylisoquinoline-1,3,4(2H)-trione* (**3h**) [14,39]: Light yellow solid, 73% yield (40 mg). Mp. 60–61 °C. Data in accordance with literature. ^1^H-NMR (CDCl_3_, 300 MHz) δ 8.33 (d, *J* = 7.9 Hz, 1H), 8.20 (d, *J* = 7.9 Hz, 1H), 7.98–7.82 (m, 2H), 4.11 (t, *J* = 7.4 Hz, 2H), 1.74–1.68 (m, 2H), 1.66–1.41 (m, 2H), 1.03 (t, *J* = 7.4 Hz, 3H). ^13^C-NMR (CDCl_3_, 100 MHz) δ 174.7, 162.1, 156.9, 135.9, 134.3, 130.8, 129.9, 129.7, 127.7, 41.0, 29.9, 20.2, 13.7. HR-MS (ESI): calcd. for [M + H]^+^ C_13_H_14_NO_3_: 232.0968, found: 232.0963.

*2-Allylisoquinoline-1,3,4(2H)-trione* (**3i**) [10,12]: Light yellow solid, 72% yield (37 mg). Mp. 180–181 °C. Data in accordance with literature. ^1^H-NMR (CDCl_3_, 400 MHz) δ 8.35 (dd, *J* = 0.7, 7.8 Hz, 1H), 8.22 (dd, *J =* 0.8, 7.7 Hz, 1H), 7.94–7.83 (m, 2H), 5.96–5.86 (m, 1H), 5.38 (d, *J =* 17 Hz, 1H), 5.26 (d, *J =* 10 Hz, 1H), 4.66 (d, *J* = 6.1 Hz, 2H). ^13^C-NMR (CDCl_3_, 75 MHz) δ 174.7, 162.1, 156.9, 136.3, 134.7, 134.5, 131.0, 130.1, 130.0, 128.1, 119.4, 43.4. HR-MS (ESI): calcd. for [M + H]^+^ C_10_H_12_NO_3_: 216.0655, found: 216.0657.

### 3.4. General Procedure for Isoquinoline-1,3,4(2H)-trione Synthesis via Staudinger Reaction

Methyl 2-(bromoacetyl)benzoate (100 mg, 0.39 mmol, 1 equiv.) and NaN_3_ (1.5 equiv.) were vigorously stirred at room temperature overnight. The suspension was diluted with MTBE and solid was filtered off. The solvent was removed under vacuum and the crude was purified by flash column chromatography on silica gel (Hexane 95/Ethyl acetate 5). To a solution of azide **4** (84 mg, 0.38 mmol, 1 equiv.) in THF/H_2_O (2 mL/200 μL) under nitrogen atmosphere, trimethylphosphine (1M in THF, 456 μL, 1.2 equiv) was added and the mixture was stirred for 4 h at room temperature. Then, the mixture was stirred under air at 50 °C overnight utilizing a rubber stopper with a needle. After evaporation of the solvent, the crude was taken up with dichloromethane and washed with water. Purification by chromatography (Ethyl acetate 3/CHCl_3_ 7) using silica gel deactivated wih 1% Et_3_N gave **3j** as yellow solid.

*Methyl 2-(2-azidoacetyl)benzoate* (**4**): Pale oil, 98% yield (84 mg). ^1^H-NMR (CDCl_3_, 400 MHz) δ 7.99 (d, *J* = 8.0 Hz, 1H), 7.61 (t, *J* = 8.0 Hz, 1H), 7.56 (*J* = 8.0 Hz, 1H), 7.30 (d, *J* = 8.0 Hz, 1H), 4.25 (s, 2H), 3.90 (s, 3H). ^13^C-NMR (CDCl_3_, 100 MHz) δ 200.2, 166.3, 140.7, 132.9, 130.4, 130.2, 127.9, 126.3, 57.8, 52.8. HR-MS (ESI): calcd. for [M + H]^+^ C_10_H_10_N_3_O_3_: 219.0644, found: 219.0638.

*Isoquinoline-1,3,4(2H)-trione* (**3j**) [12,21,38]: Yellow solid, 62% yield (41 mg). Mp. 220–221 °C. Data in accordance with literature. ^1^H-NMR (DMSO-*d_6_*, 400 MHz) δ 11.98 (br, 1H), 8.14 (d, *J* = 7.9 Hz, 1H), 8.07 (d, *J* = 7.6 Hz, 1H), 7.95–7.88 (m, 2H). ^13^C-NMR (DMSO-*d_6_*, 75 MHz) δ 174.8, 162.7, 157.4, 136.5, 134.8, 131.2, 130.1, 129.7, 128.1. HR-MS (ESI): calcd. for [M + H]^+^ C_9_H_6_NO_3_: 176.0342, found: 176.0346.

## 4. Conclusions

In conclusion, we report simple and synthetically useful protocols for the preparation of 2,3-dihydroisoquinoline-1,3,4-triones by one-pot cascade reactions of methyl 2-bromoacetyl)benzoate with primary amines followed by in situ oxidation promoted by air, without the use of any other reagent or catalyst. This transformation proved to be of general applicability with respect to aliphatic or benzylic amines, while aromatic amines like aniline lead to decomposition products. A suitable modification of the original protocol utilizing a Staudinger reaction, allowed the synthesis of the NH-unsubstituted caspase inhibitor trione. On the basis of the present work, other studies are in course in order to analyze the applicability of the concepts herein developed and in particular of the metal-free air oxidation on related multifunctional substrates.

## Figures and Tables

**Table 1:**
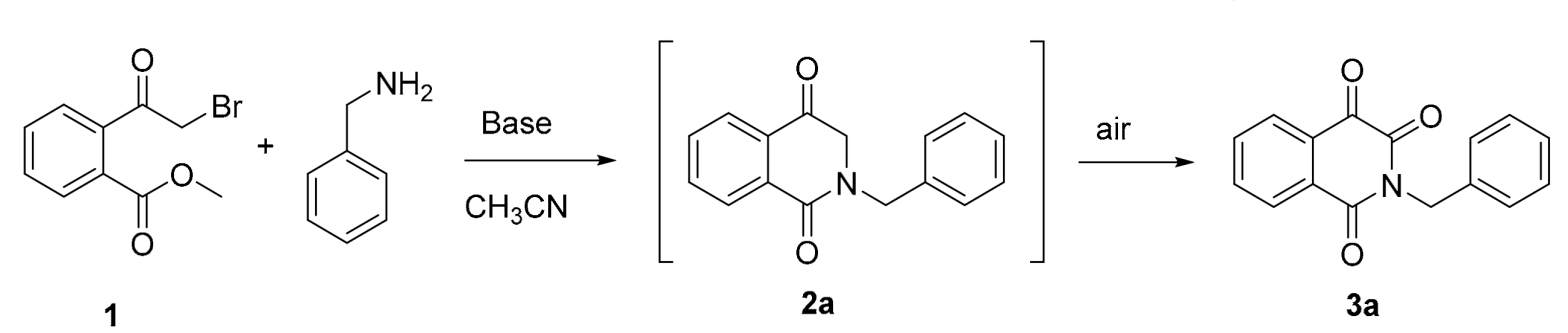
Preliminary reaction conditions screening.

Entry	Base (1 eq)	T (°C)	Time (h)	Yield 3a ^a^
**1**	-	r.t.	3	--
**2**	-	r.t.	18	--
**3**	DIPEA	r.t	18	18% ^b^
**4**	DIPEA	50	3	53%
**5**	DIPEA	50	18	71%
**6** ^c^	DIPEA	50	18	10%
**7**	Et_3_N	50	18	50%
**8**	NaHCO_3_	r.t	18	26%
**9**	NaHCO_3_	50	3	41%
**10**	K_2_CO_3_	50	4	40%
**11**	K_2_CO_3_	50	18	Dec.

^a^ Yields refer to chromatographically pure compounds. ^b^ 80% of **1** was recovered. ^c^ Reaction was performed under nitrogen.

**Table 2:**
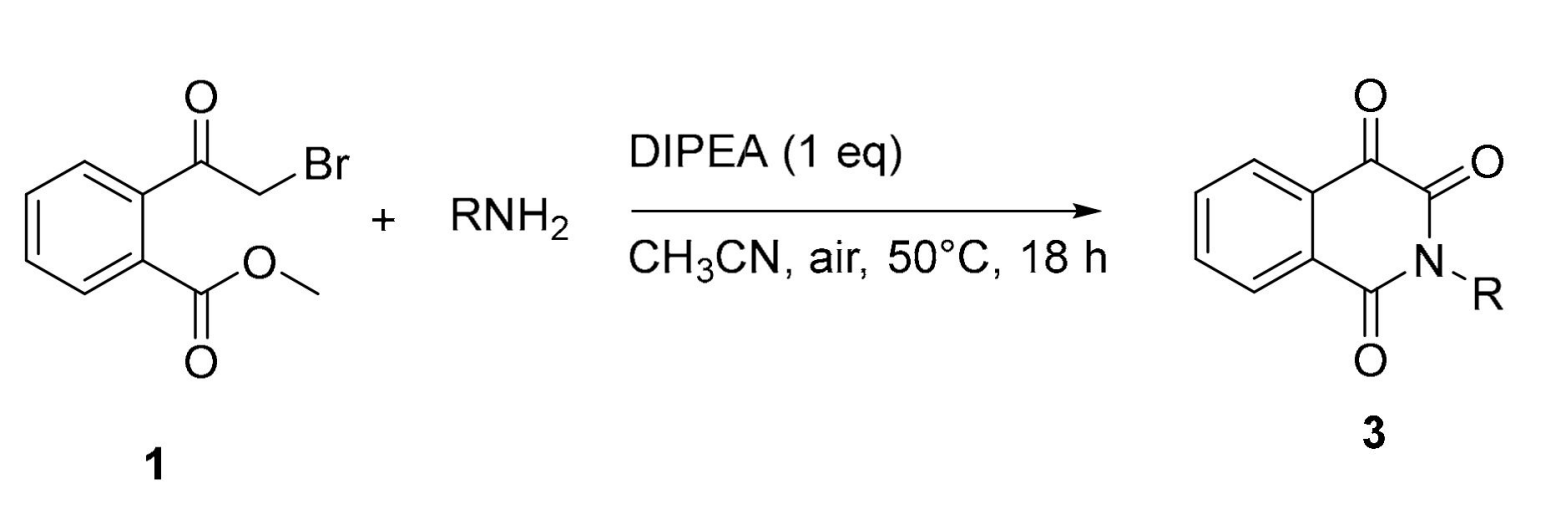
Substrate scope.

Entry	R	3	Yield % ^a^
**1**	-CH_2_Ph	**(3a)**	71%
**2**	4-Cl-CH_2_Ph	**(3b)**	73%
**3**	4-MeO-CH_2_Ph	**(3c)**	75%
**4**	2-F-CH_2_Ph	**(3d)**	69%
**5**	3-NO_2_-CH_2_Ph	**(3e)**	69%
**6**	-CH_3_	**(3f)**	72%
**7**	-CH_2_CH_3_	**(3g)**	70%
**8** **9**	-(CH_2_)_3_CH_3_ -CH_2_CH=CH_2_	**(3h)** **(3i)**	73% 72%
**10**	Ph		--

^a^ Yields refer to chromatographically pure compounds.

## Supplementary Materials

The following are available online at https://www.mdpi.com/1420-3049/24/11/2177/s1, Figure S1: The X-ray molecular structure of compound 3a and X-Ray data and NMR spectra of all the compounds.
