# Supplementary material for: Metal-Free Air Oxidation in a Convenient Cascade Approach for the Access to Isoquinoline-1,3,4(2H)-triones
Source: Molecules. 2019 Jun 10;24(11):2177. doi: 10.3390/molecules24112177 (PMC6600517; doi:10.3390/molecules24112177)
Supplement: Supplementary file 1 [file molecules-24-02177-s001.pdf]

Supplementary Materials

Metal-free air oxidation in a convenient cascade approach for the access to Isoquinoline-1,3,4(2*H*)-triones

Antonia Di Mola[1*], Consiglia Tedesco[1] and Antonio Massa[1*]

Dipartimento di Chimica e Biologia "A. Zambelli", Università degli studi di Salerno, Via Giovanni Paolo II, 84084-Fisciano (SA), Italy.

* Correspondence: amassa@unisa.it., toniadimola@libero.it, Phone: +39-089969565.

Contents

1. X-Ray data 2-benzylisoquinoline-1,3,4(2H)-trione (**3a**)....................2
2. NMR Spectra of compounds 4

Single crystals suitable for X-ray diffraction studies of compound **3a** were obtained by recrystallization from $CHCl_3$/hexane solution. The X-ray molecular structure is shown in Figure S1. Bond distances and angles are similar to those of analogous iso-quinoline-1,3,4-trione compounds (R. M. Ghalib, C. S. Chidan Kumar, R. Hashim, O. Sulaiman and H.-K. Fun Acta Cryst. E , **2015**, 71, o6-o7).

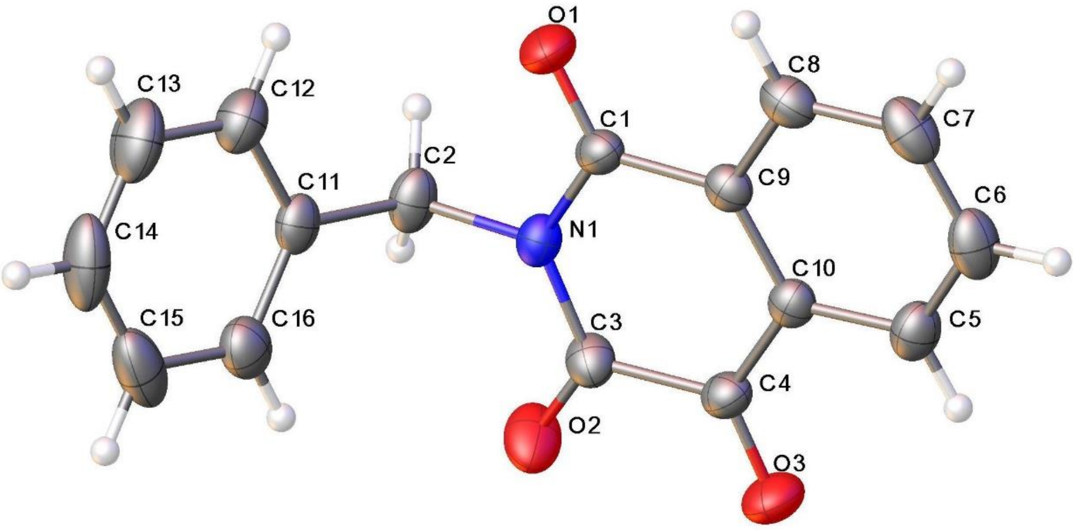

Figure 1. The X-ray molecular structure of compound **3a**.

X-ray crystallography

Colourless prismatic single crystals of compound **3a** suitable for X-ray diffraction analysis were obtained by $CHCl_3$/hexane solution.

A suitable crystal (0.44 mm × 0.35 mm × 0.27 mm)

was glued on a glass fiber and measured at room temperature at room temperature with a Bruker D8 QUEST diffractometer equipped with a PHOTON100 detector using Cu*Kα* radiation (λ= 1.54178 Å). Indexing was performed using APEX3.[1] Data integration and reduction were performed using SAINT.[1] Absorption correction was performed by multi-scan method in SADABS.[1]

The structure was solved by Direct Methods using SIR2014[2] and refined by means of full matrix least-squares based on F^2 using the program SHELXL.[3] 18760 reflections were collected, resulting in 2328 independent reflections (R_{int} = 0.0199, R_{sigma} = 0.0123).

Non-hydrogen atoms were refined anisotropically, hydrogen atoms were positioned geometrically and included in structure factors calculations, but not refined. 181 refinable parameters were finally considered, final disagreement R indices (all data) are: R =0.0496,

wR_2 = 0.1249. The maximum and minimum residual densities were respectively 0.18/-0.20 eÅ^{-3}.

Crystal structures were drawn using OLEX2.[4]

Crystal data. Formula: $C_{16}H_{11}NO_3$, Formula weight: 265.26, Crystal system: monoclinic, Space group: $P2_1/n$, a = 13.148(2) Å , b = 7.3323(8) Å, c= 13.8239(12) Å, β107.179(9)° V= /Å1273.2(3) Å^3, Z= 4, ρ_{calc}=1.384 g/cm^3, μ= 0.795mm^{-1}, F(000)=552.0

Supporting Information Available: Diffraction data in cif format of compounds **3a**

CCDC 1915668

References

[1] CrystalClear, Crystal Structure Analysis Package, Rigaku-Molecular Structure Corp.

[2] APEX3, SAINT, SADABS. Bruker AXS Inc., Madison, Wisconsin, USA. Bruker AXS Inc., Madison, Wisconsin, USA, **2012**.

[3] Burla, M. C.; Caliandro, R.; Carrozzini, B.; Cascarano, G. L.; Cuocci, C.; Giacovazzo, C.; Mallamo, M.; Mazzone, A.; Polidori, G. Crystal structure determination and refinement via SIR2014. *J. Appl. Cryst.* **2015**, *48*, 306-309.

[4] Sheldrick, G. M. Crystal structure refinement with SHELXL. *Acta Cryst.* **2015**, *C71*, 3–8

[5] Dolomanov, O. V.; Bourhis, L. J.; Gildea, R. J.; Howard, J. A. K.; Puschmann, H. OLEX2: a complete structure solution, refinement and analysis program. *J. Appl. Cryst.* **2009**, *42*, 339–341.

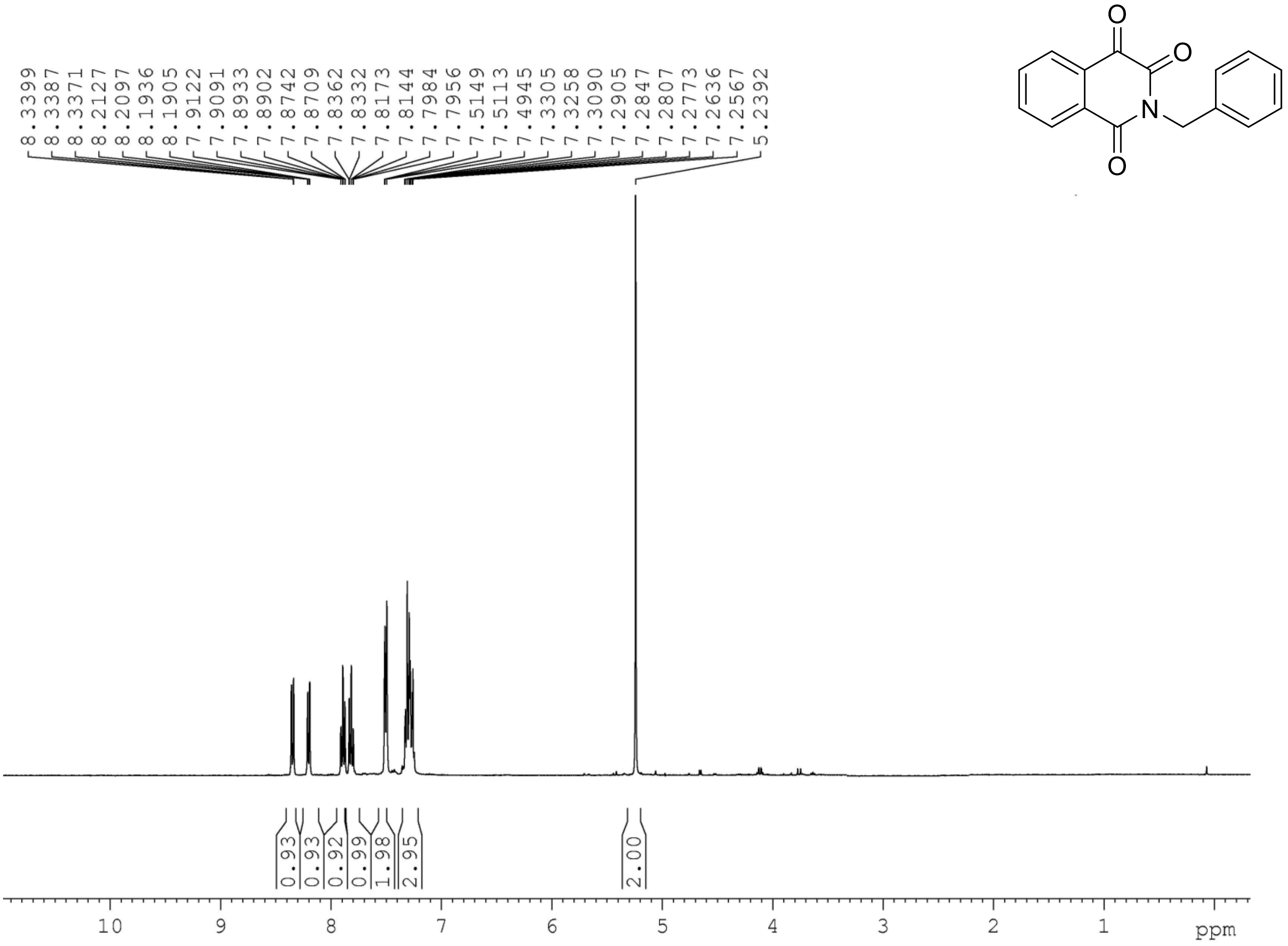
O
O
N
O
8.3399
8.3387
8.3371
8.2127
8.2097
8.1936
8.1905
7.9122
7.9091
7.8933
7.8902
7.8742
7.8709
7.8362
7.8332
7.8173
7.8144
7.7984
7.7956
7.5149
7.5113
7.4945
7.3305
7.3258
7.3090
7.2905
7.2847
7.2807
7.2773
7.2636
7.2567
5.2392
0.93
0.93
0.92
0.99
1.98
2.95
2.00
10
9
8
7
6
5
4
3
2
1
ppm

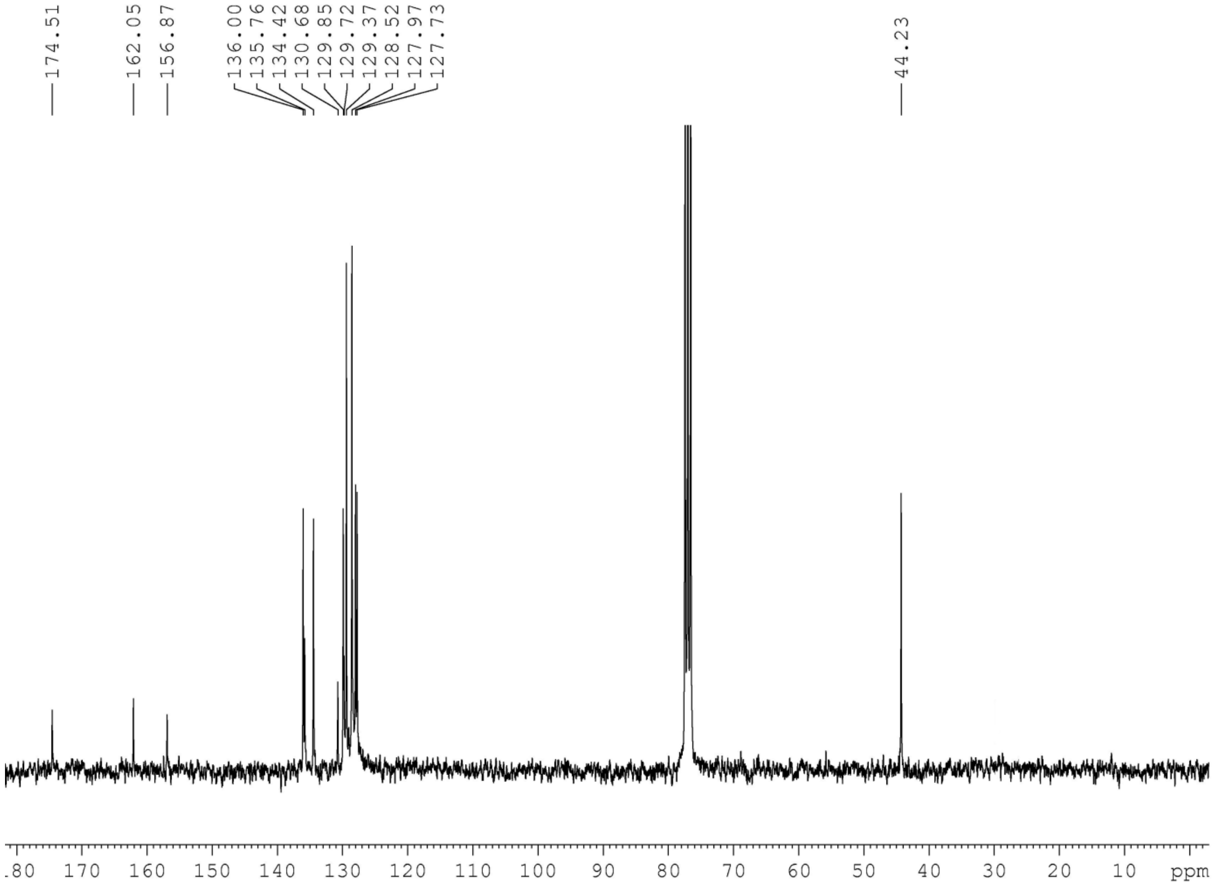
174.51
162.05
156.87
136.00
135.76
134.42
130.68
129.85
129.72
129.37
128.52
127.97
127.73
44.23
.80
170
160
150
140
130
120
110
100
90
80
70
60
50
40
30
20
10
ppm

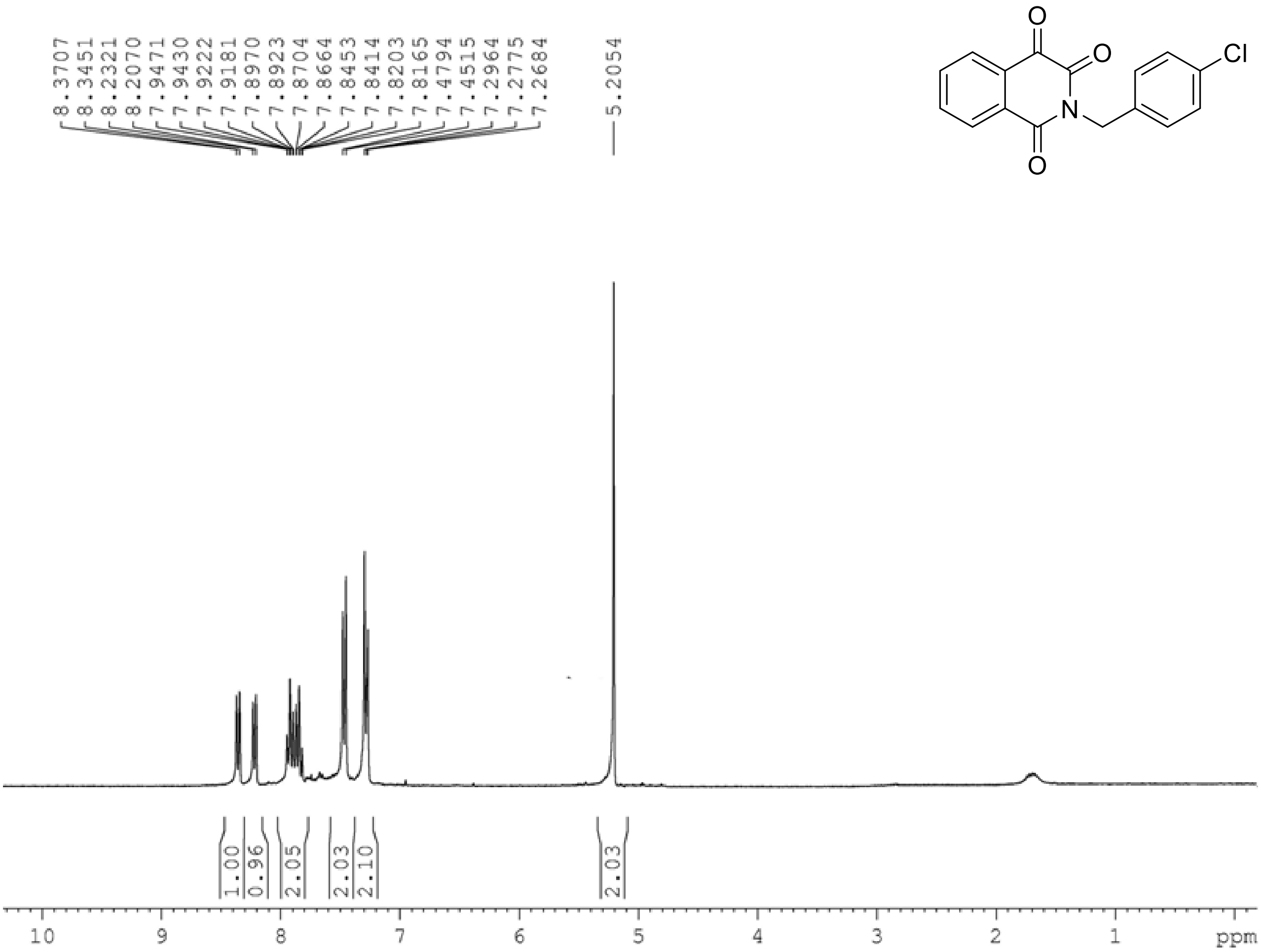
8.3707 8.3451 8.2321 8.2070 7.9471 7.9430 7.9222 7.9181 7.8970 7.8923 7.8704 7.8664 7.8453 7.8414 7.8203 7.8165 7.4794 7.4515 7.2964 7.2775 7.2684
5.2054
O
O
N
Cl
O
1.00
0.96
2.05
2.03
2.10
2.03
10 9 8 7 6 5 4 3 2 1 ppm

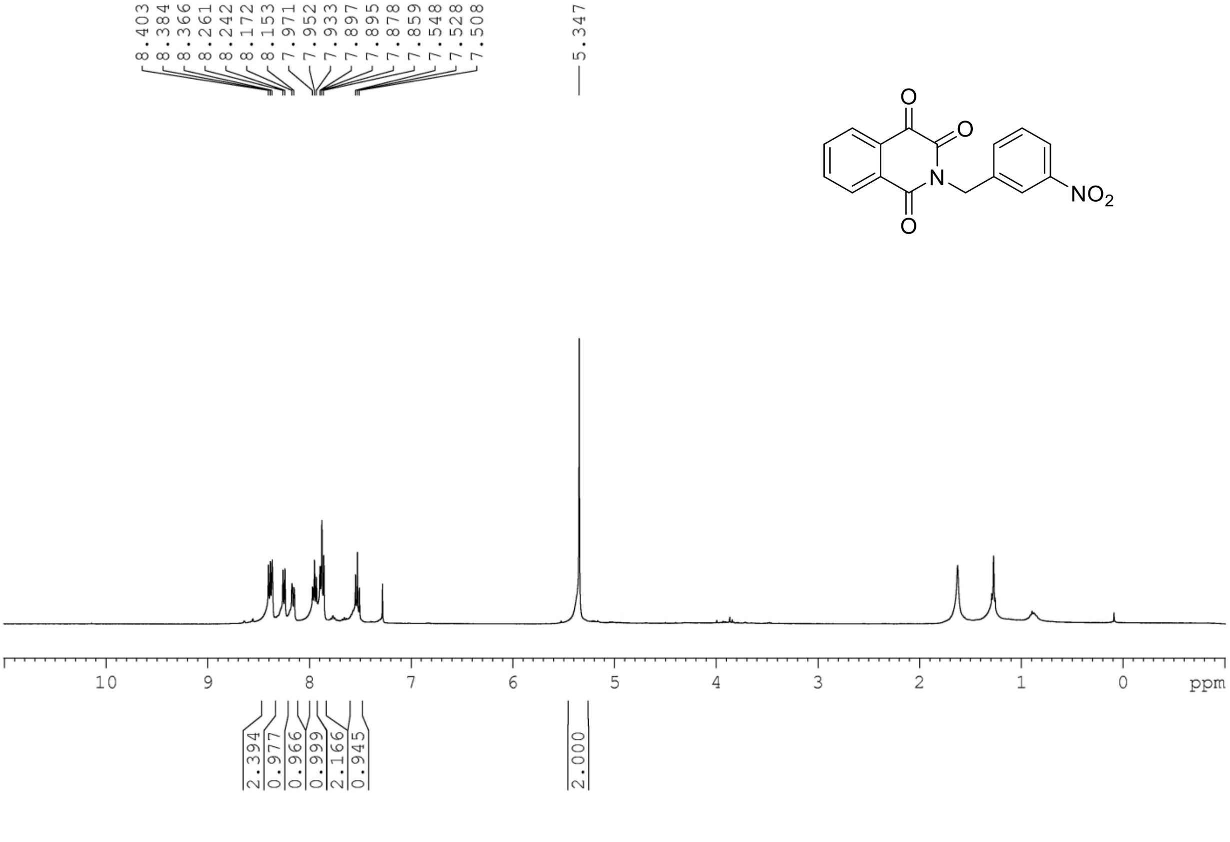
8.403 8.384 8.366 8.261 8.242 8.172 8.153 7.971 7.952 7.933 7.897 7.895 7.878 7.859 7.548 7.528 7.508
5.347
O
O
N
NO2
O
10 9 8 7 6 5 4 3 2 1 0 ppm
2.394
0.977
0.966
0.999
2.166
0.945
2.000

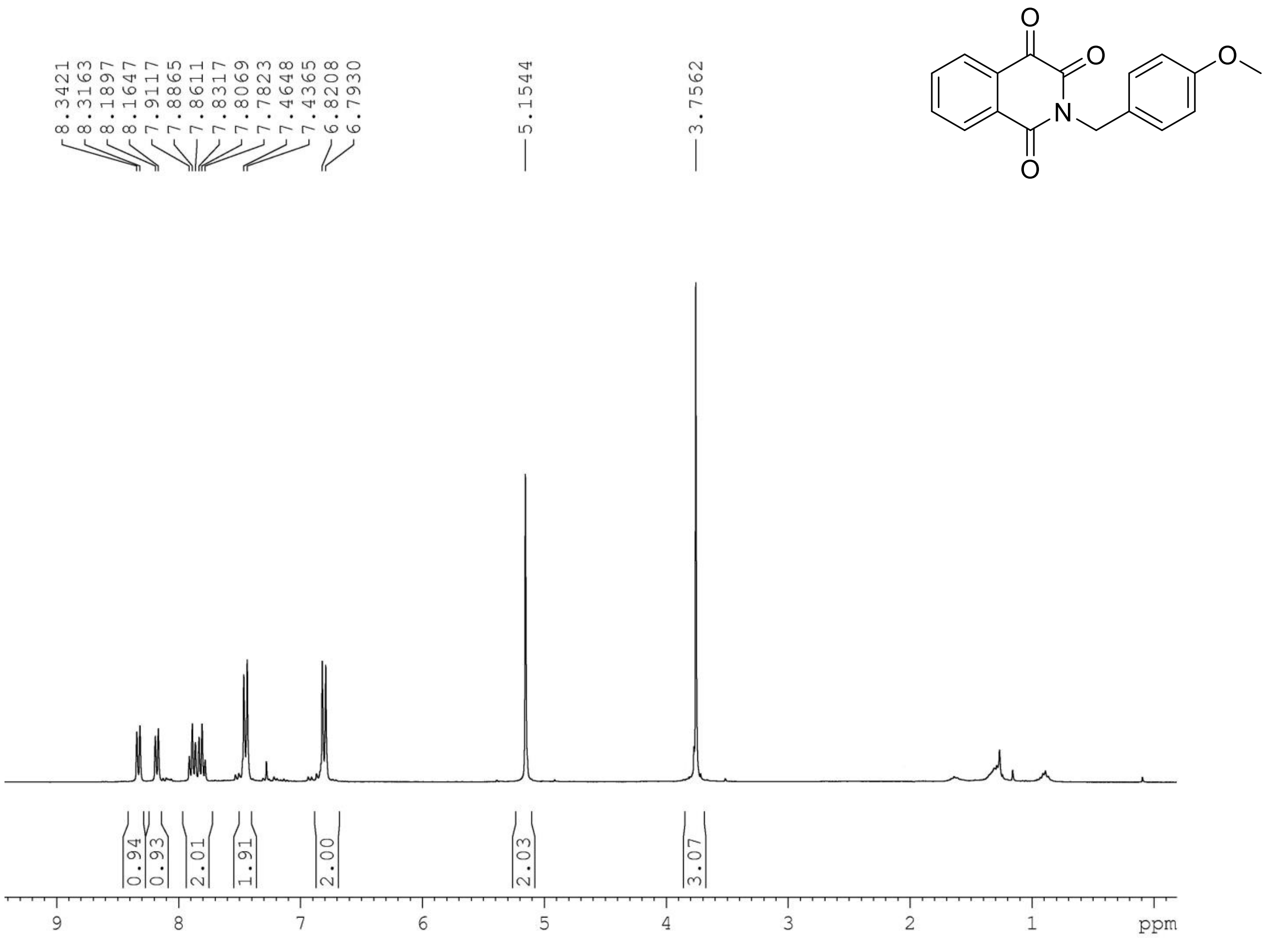
O
O
N
O
O
8.3421
8.3163
8.1897
8.1647
7.9117
7.8865
7.8611
7.8317
7.8069
7.7823
7.4648
7.4365
6.8208
6.7930
5.1544
3.7562
0.94
0.93
2.01
1.91
2.00
2.03
3.07
9
8
7
6
5
4
3
2
1
ppm

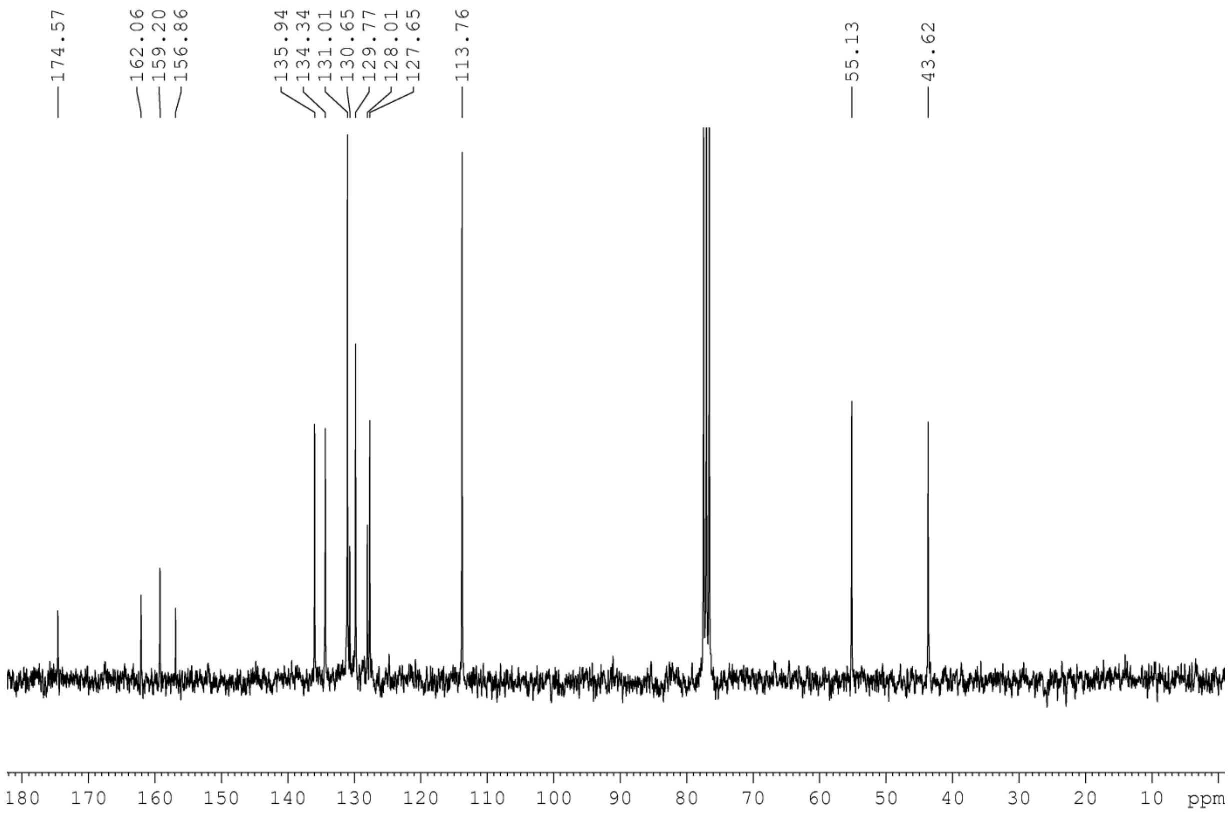
174.57
162.06
159.20
156.86
135.94
134.34
131.01
130.65
129.77
128.01
127.65
113.76
55.13
43.62
180
170
160
150
140
130
120
110
100
90
80
70
60
50
40
30
20
10
ppm

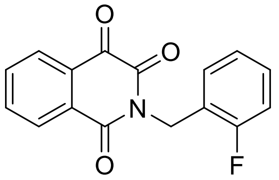
O
O
N
O
F

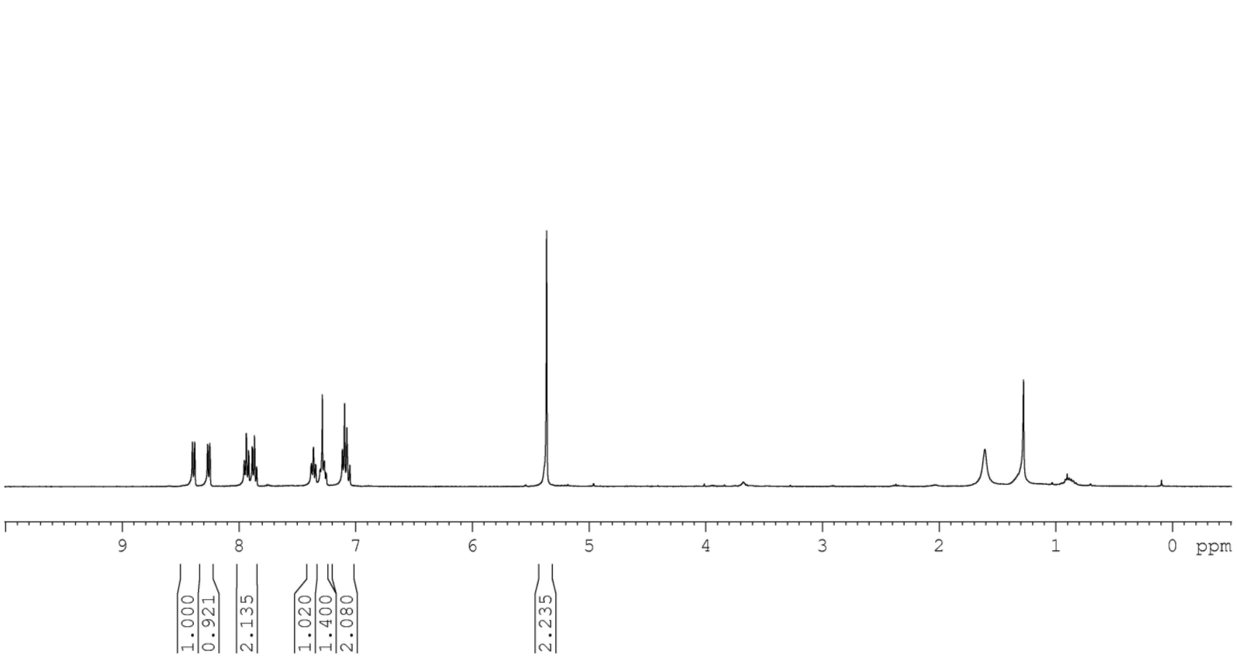
8.265
8.248
8.246
7.954
7.951
7.935
7.932
7.916
7.913
7.885
7.882
7.866
7.863
7.847
7.844
7.377
7.359
7.342
7.304
7.299
7.283
7.271
7.266
7.252
7.110
7.093
7.073
7.049
5.361
9
8
7
6
5
4
3
2
1
0 ppm
1.000
0.921
2.135
1.020
1.400
2.080
2.235

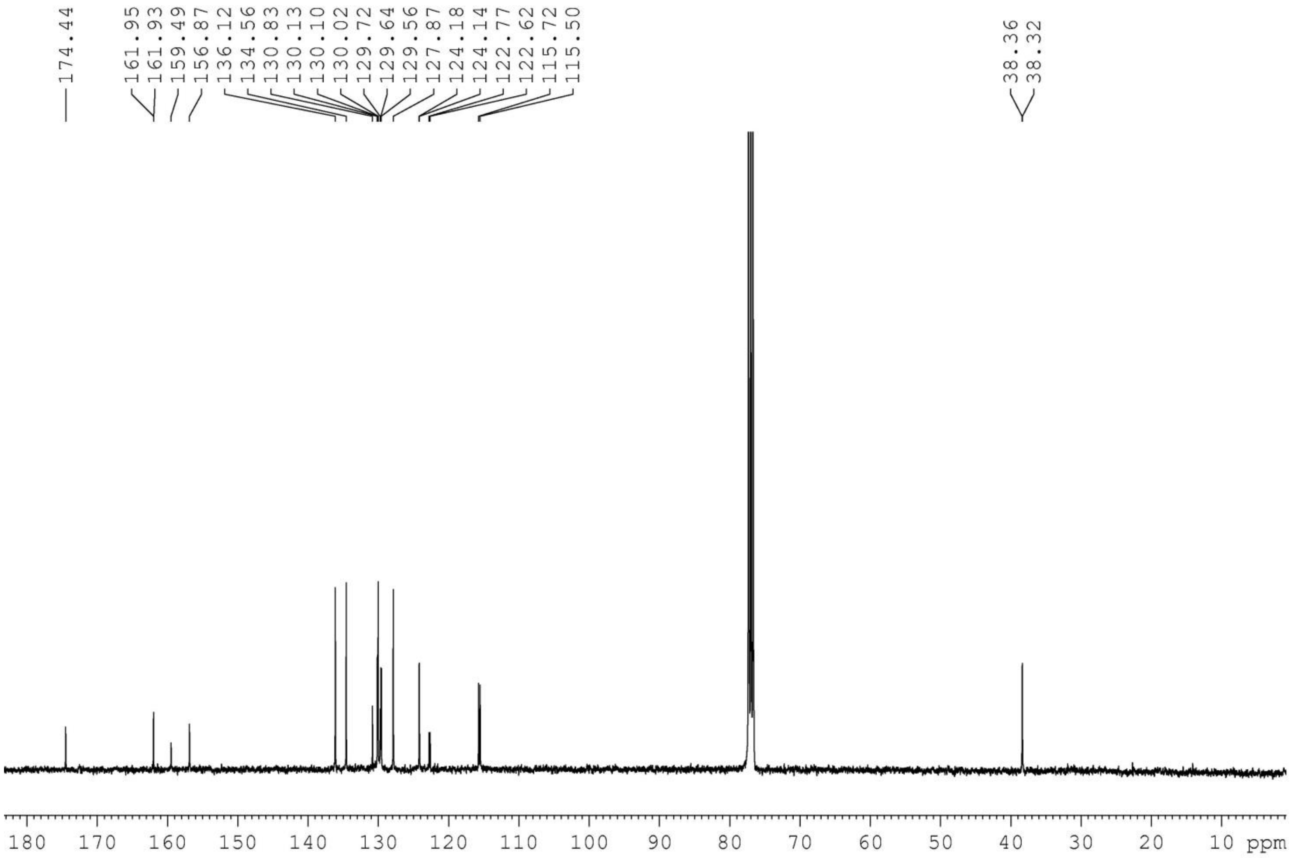
174.44
161.95
161.93
159.49
156.87
136.12
134.56
130.83
130.13
130.10
130.02
129.72
129.64
129.56
127.87
124.18
124.14
122.77
122.62
115.72
115.50
38.36
38.32
180
170
160
150
140
130
120
110
100
90
80
70
60
50
40
30
20
10 ppm

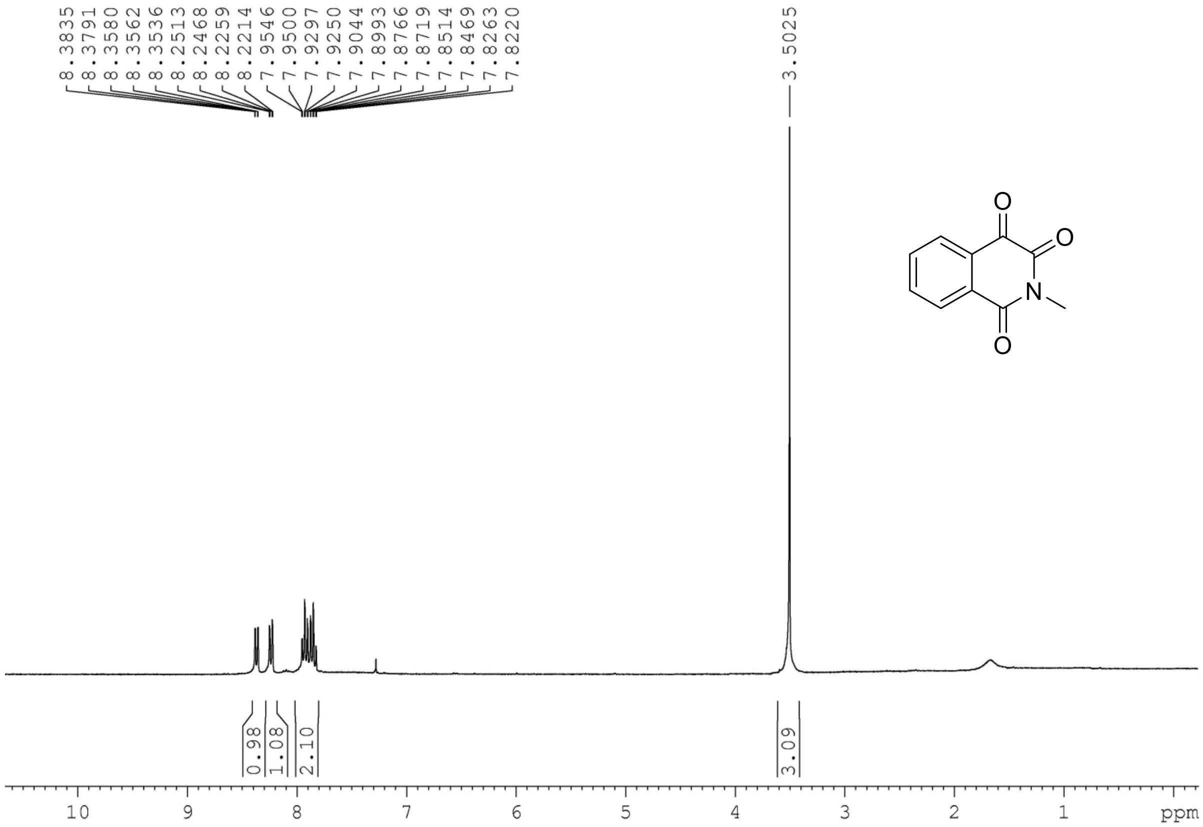
8.3835
8.3791
8.3580
8.3562
8.3536
8.2513
8.2468
8.2259
8.2214
7.9546
7.9500
7.9297
7.9250
7.9044
7.8993
7.8766
7.8719
7.8514
7.8469
7.8263
7.8220
3.5025
O
O
N
O
0.98
1.08
2.10
3.09
10
9
8
7
6
5
4
3
2
1
ppm

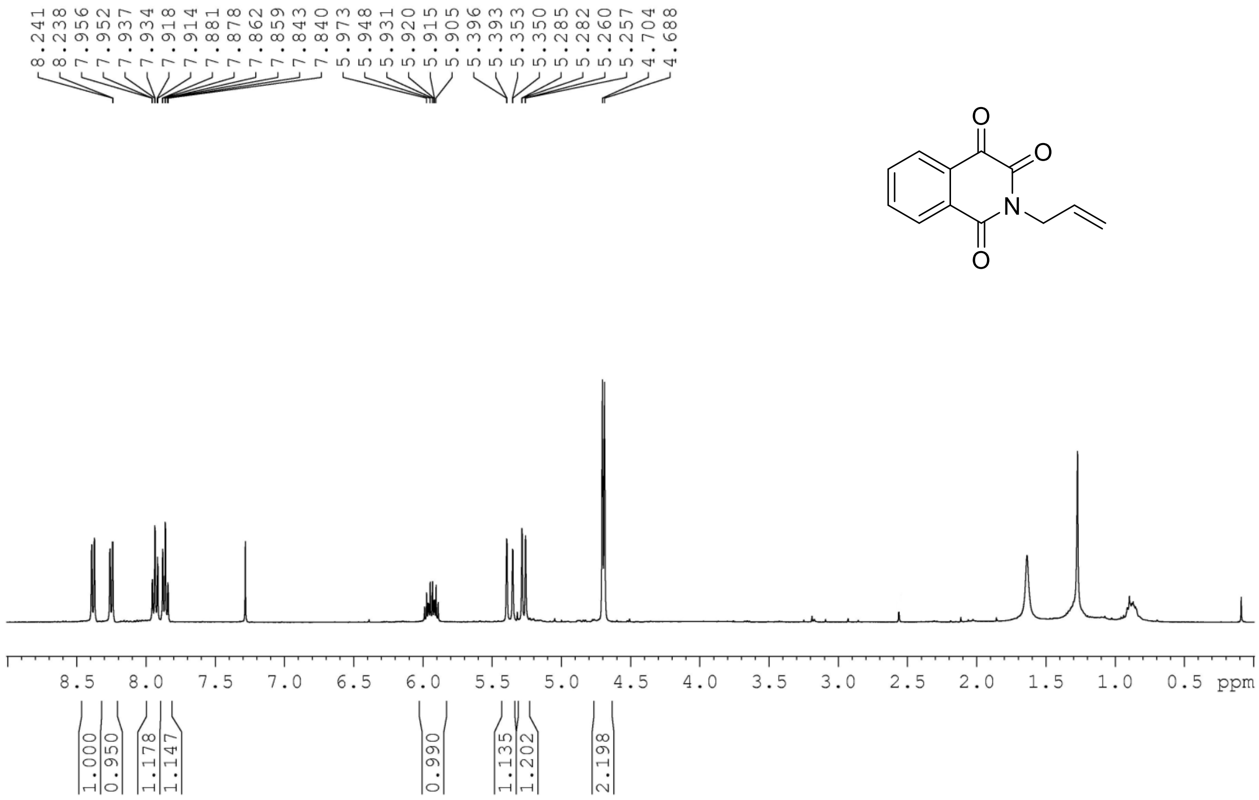
8.241
8.238
7.956
7.952
7.937
7.934
7.918
7.914
7.881
7.878
7.862
7.859
7.843
7.840
5.973
5.948
5.931
5.920
5.915
5.905
5.396
5.393
5.353
5.350
5.285
5.282
5.260
5.257
4.704
4.688
O
O
N
O
8.5
8.0
7.5
7.0
6.5
6.0
5.5
5.0
4.5
4.0
3.5
3.0
2.5
2.0
1.5
1.0
0.5 ppm
1.000
0.950
1.178
1.147
0.990
1.135
1.202
2.198

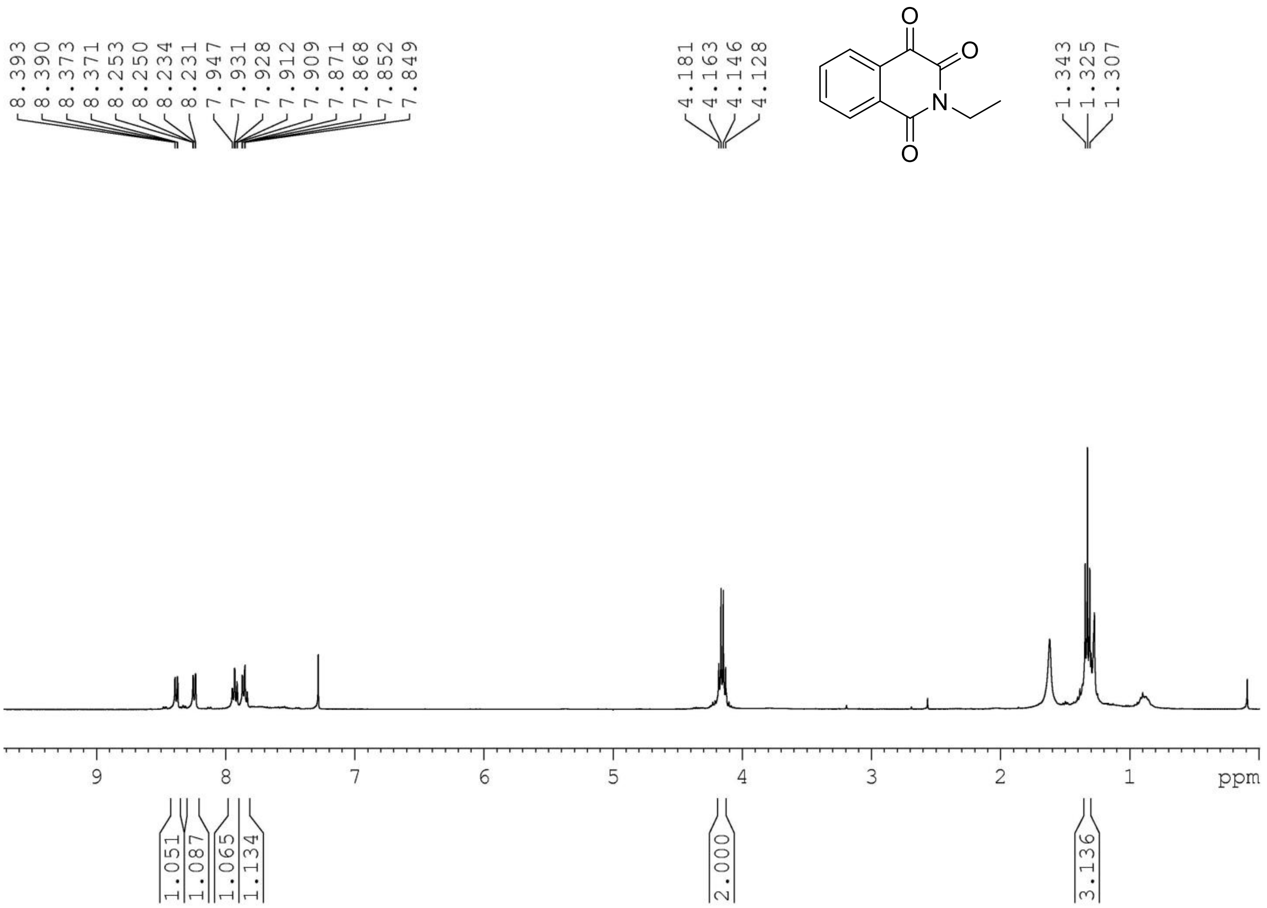
8.393
8.390
8.373
8.371
8.253
8.250
8.234
8.231
7.947
7.931
7.928
7.912
7.909
7.871
7.868
7.852
7.849
4.181
4.163
4.146
4.128
O
O
N
O
1.343
1.325
1.307
9
8
7
6
5
4
3
2
1
ppm
1.051
1.087
1.065
1.134
2.000
3.136

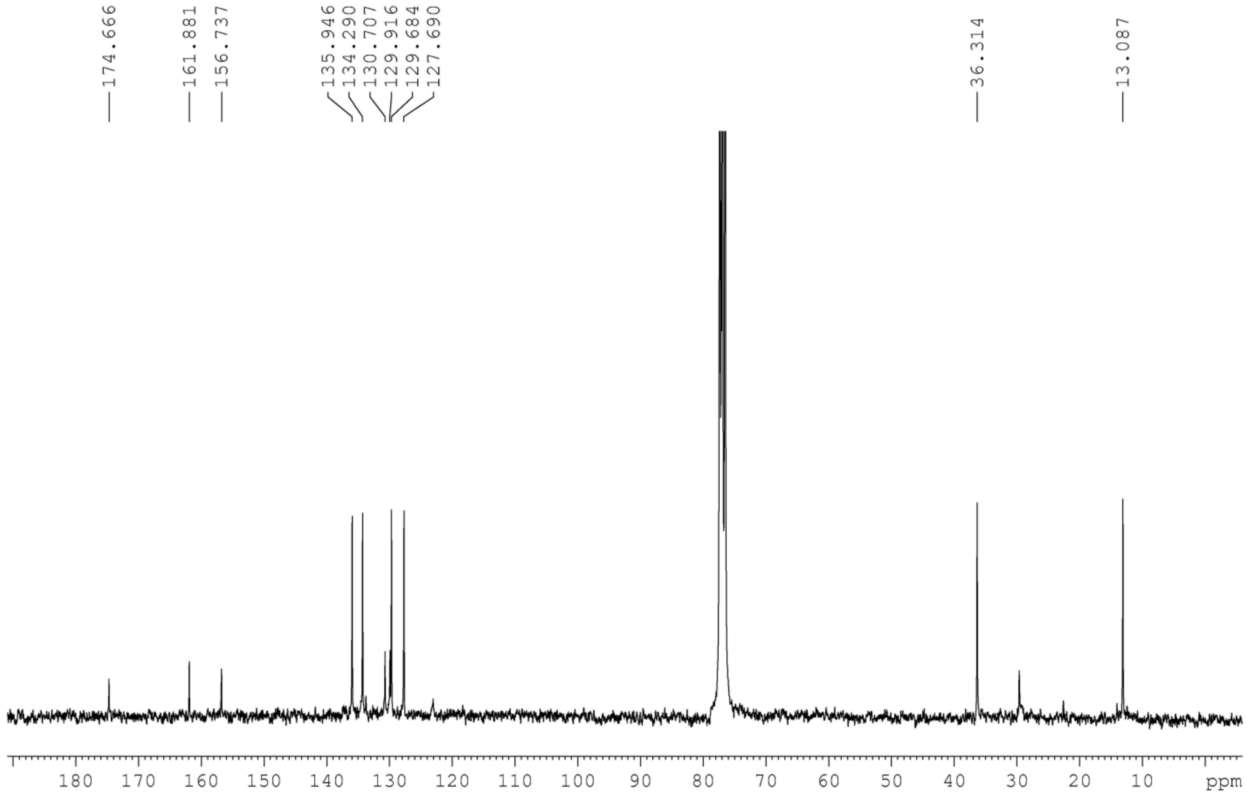
174.666
161.881
156.737
135.946
134.290
130.707
129.916
129.684
127.690
36.314
13.087
180
170
160
150
140
130
120
110
100
90
80
70
60
50
40
30
20
10
ppm

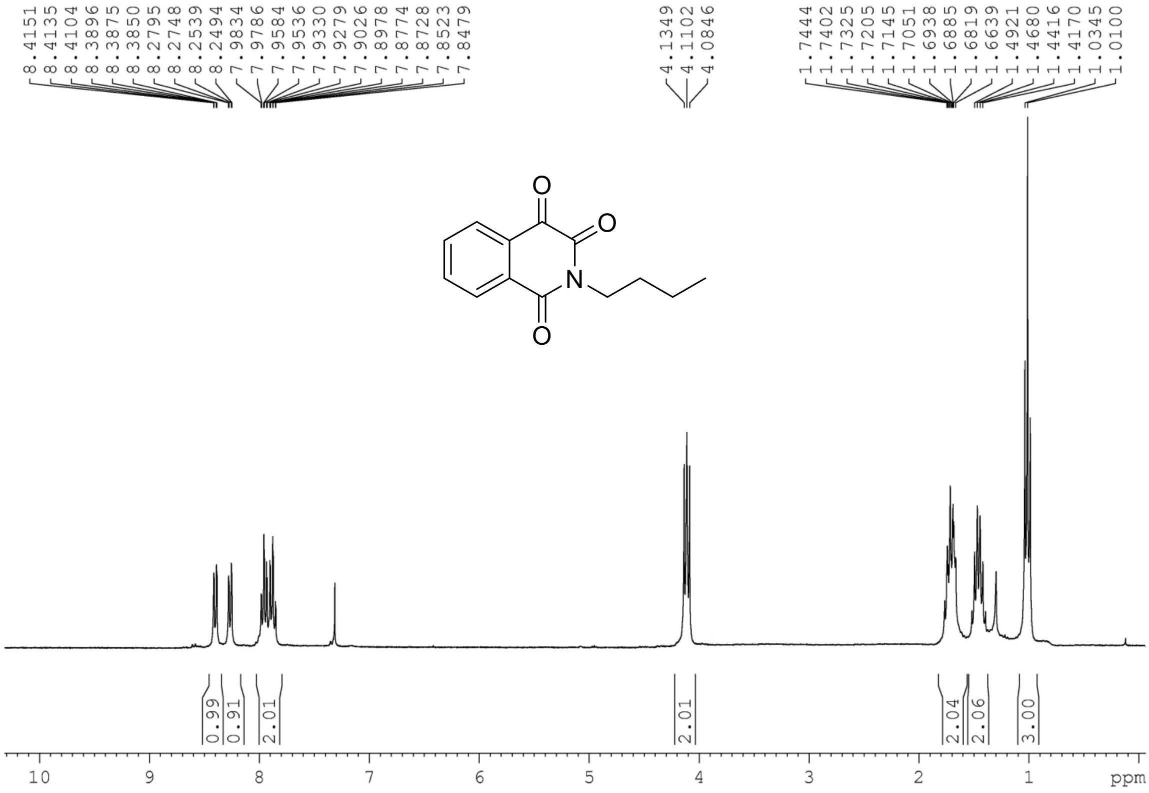
8.4151
8.4135
8.4104
8.3896
8.3875
8.3850
8.2795
8.2748
8.2539
8.2494
7.9834
7.9786
7.9584
7.9536
7.9330
7.9279
7.9026
7.8978
7.8774
7.8728
7.8523
7.8479
4.1349
4.1102
4.0846
1.7444
1.7402
1.7325
1.7205
1.7145
1.7051
1.6938
1.6885
1.6819
1.6639
1.4921
1.4680
1.4416
1.4170
1.0345
1.0100
O
O
N
O
0.99
0.91
2.01
2.01
2.04
2.06
3.00
10
9
8
7
6
5
4
3
2
1
ppm

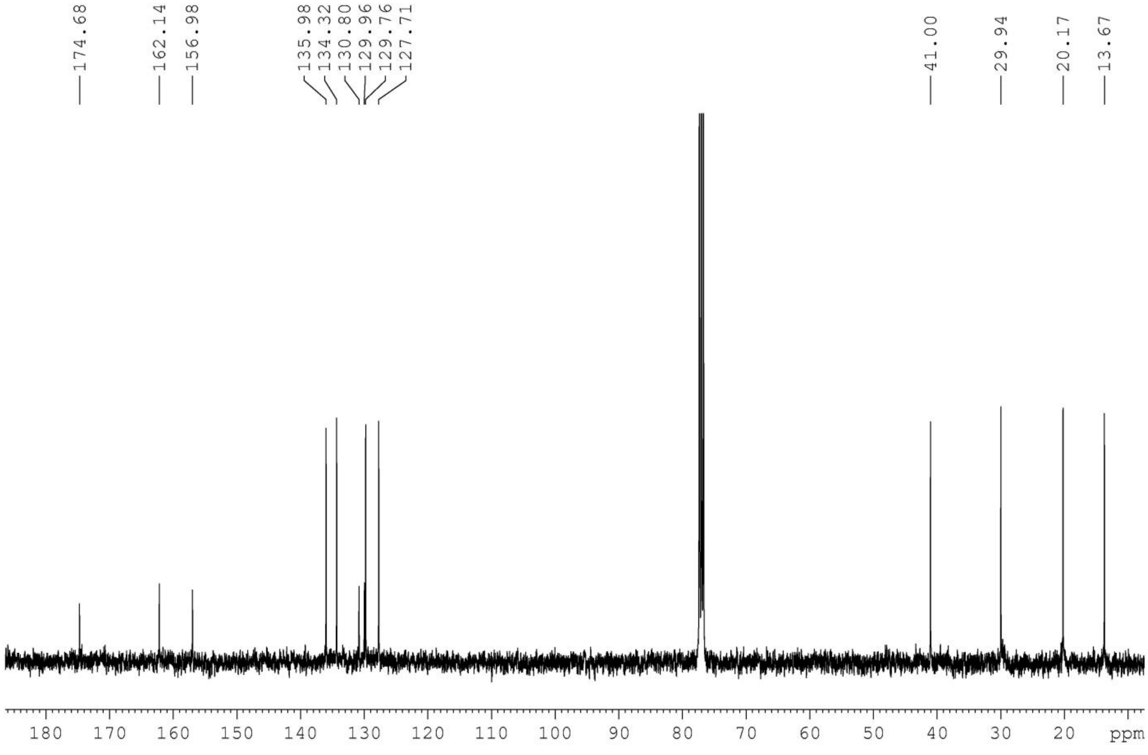
174.68
162.14
156.98
135.98
134.32
130.80
129.96
129.76
127.71
41.00
29.94
20.17
13.67
180
170
160
150
140
130
120
110
100
90
80
70
60
50
40
30
20
ppm

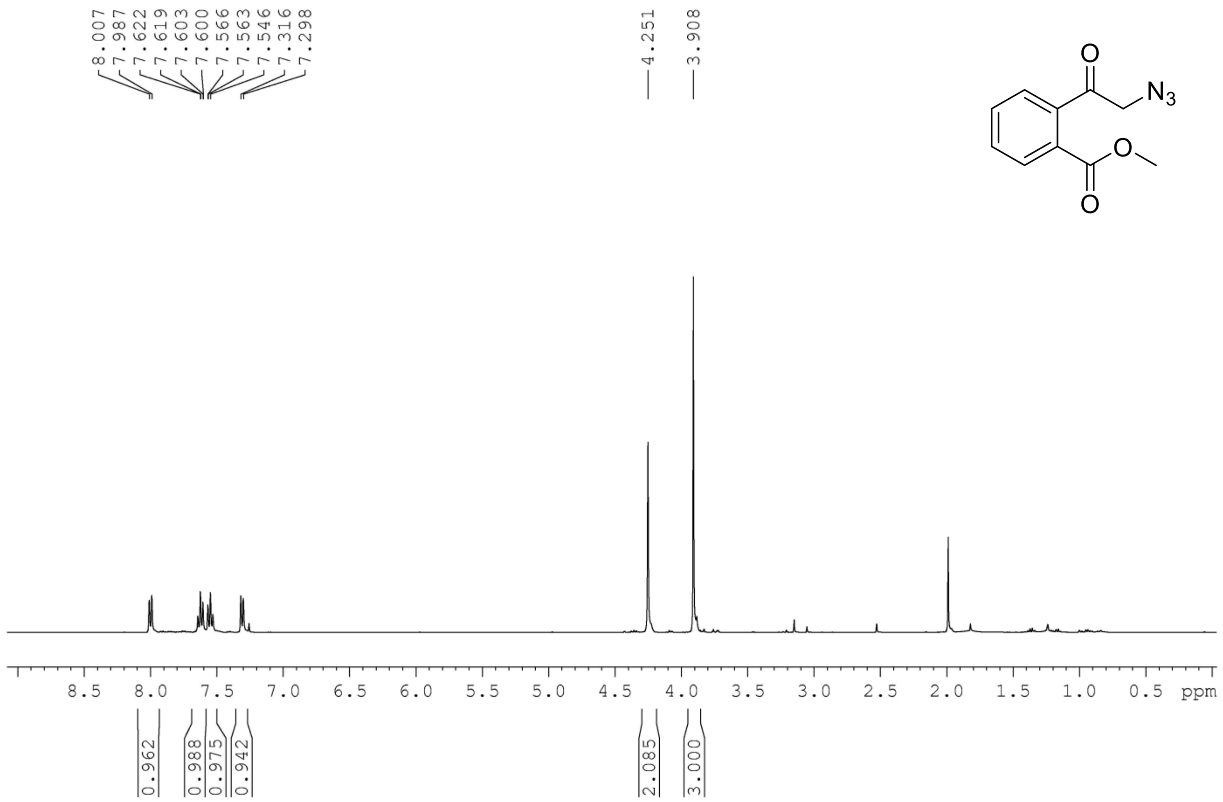
O
N3
O
O
8.007
7.987
7.622
7.619
7.603
7.600
7.566
7.563
7.546
7.316
7.298
4.251
3.908
8.5 8.0 7.5 7.0 6.5 6.0 5.5 5.0 4.5 4.0 3.5 3.0 2.5 2.0 1.5 1.0 0.5 ppm
0.962
0.988
0.975
0.942
2.085
3.000

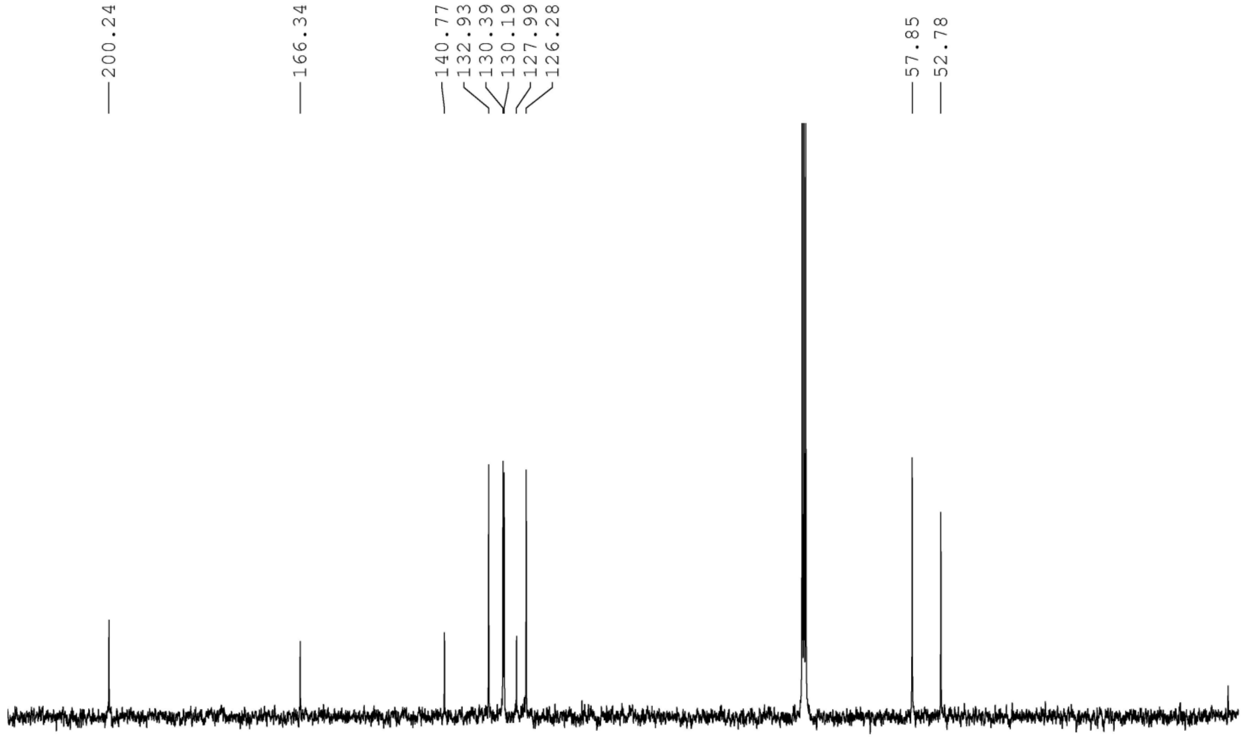
200.24
166.34
140.77
132.93
130.39
130.19
127.99
126.28
57.85
52.78
200 180 160 140 120 100 80 60 40 20 ppm

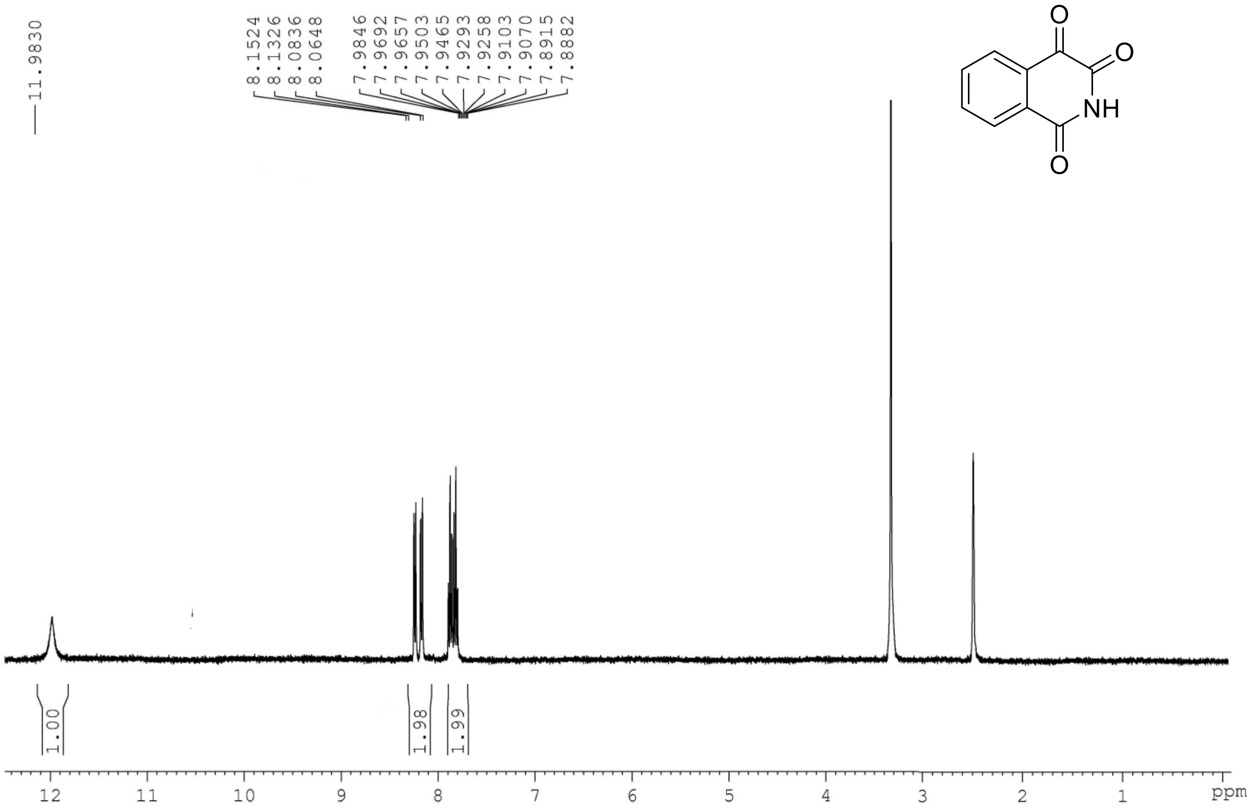
11.9830
8.1524
8.1326
8.0836
8.0648
7.9846
7.9692
7.9657
7.9503
7.9465
7.9293
7.9258
7.9103
7.9070
7.8915
7.8882
O
O
NH
O
1.00
1.98
1.99
12 11 10 9 8 7 6 5 4 3 2 1 ppm

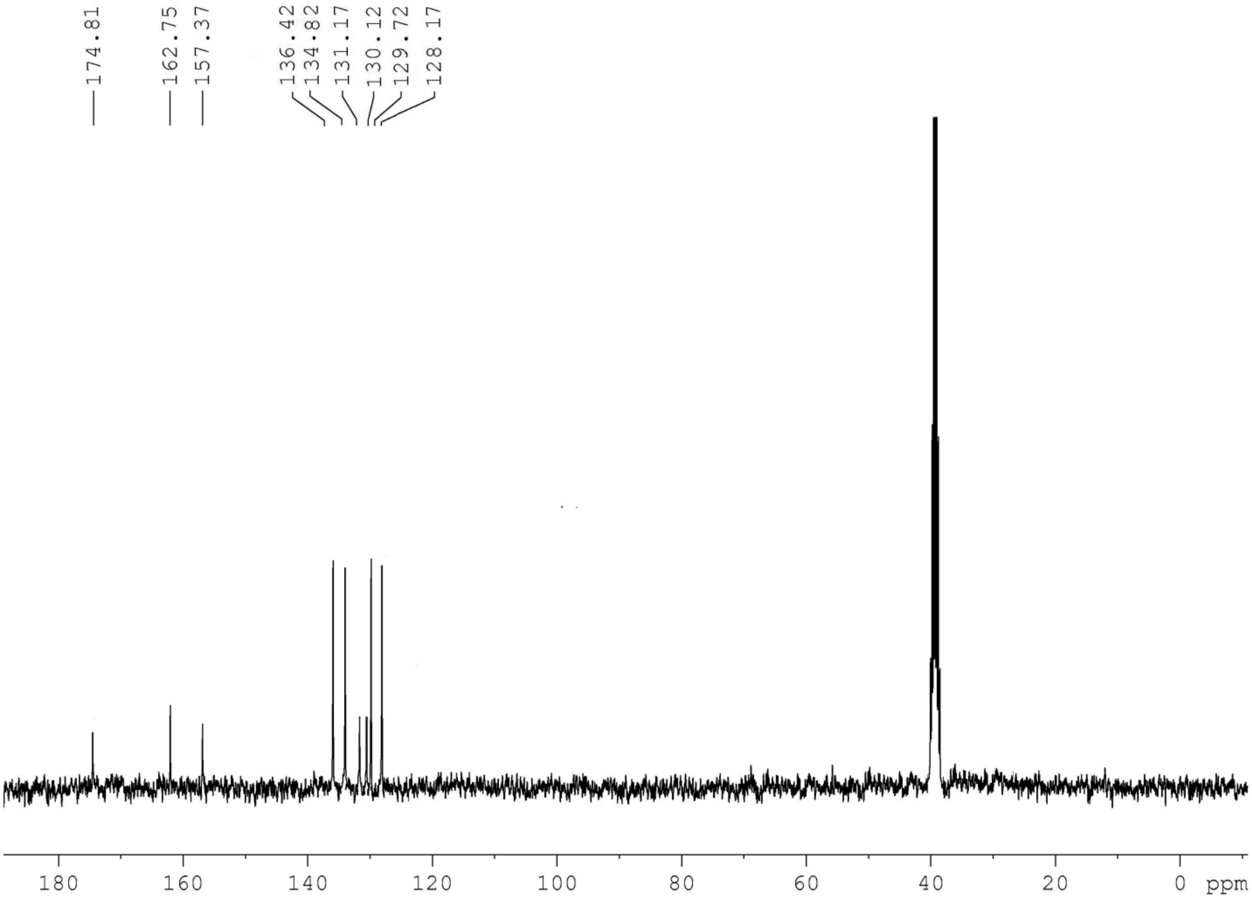
174.81
162.75
157.37
136.42
134.82
131.17
130.12
129.72
128.17
180 160 140 120 100 80 60 40 20 0 ppm
